# Solid-state MAS NMR, TEM, and TGA studies of structural hydroxyl groups and water in nanocrystalline apatites prepared by dry milling

**DOI:** 10.1007/s11051-013-1868-y

**Published:** 2013-07-30

**Authors:** Lukasz Pajchel, Waclaw Kolodziejski

**Affiliations:** Department of Inorganic and Analytical Chemistry, Faculty of Pharmacy, Medical University of Warsaw, ul. Banacha 1, 02-097 Warsaw, Poland

**Keywords:** Apatite, Milling, Nanocrystals, Hydroxyl groups, Structural water, NMR

## Abstract

A series of nanocrystalline calcium hydroxyapatites was prepared by dry milling and characterized using proton and ^31^P MAS NMR, TEM, and TGA methods. The samples contained stubby rod-shaped crystals, which length and width varied in the 130–30 and 95–20 nm ranges, respectively. It was confirmed that concentration of structural hydroxyl groups in nanocrystalline apatites decreases with the decreasing crystal size. In the series of the studied apatites, the decrease was from 86 to ca. 50 % in reference to stoichiometric apatite. Water was found in the surface hydrated layer and in the *c*-axis channels, in which compartments existed as adsorbed and structural, respectively. Molecules of the adsorbed water were capable of moving from the crystal surface into the lattice *c*-axis channels of apatite. This process introduced considerable structural disorder within and around those channels and reduced the content of the structural hydroxyl groups, particularly in the region underneath the apatite crystal surface.

**Electronic supplementary material:**

The online version of this article (doi:10.1007/s11051-013-1868-y) contains supplementary material, which is available to authorized users.

## Introduction

Hydroxyapatites (HA) are minerals of great geological, biological, and medical importance (Aoki 1991; Dorozhkin 2009, 2010; Eichert et al. 2009; Elliott 1994; LeGeros 1991; Olszta et al. 2007). Stoichiometric calcium hydroxyapatite, Ca_10_(PO_4_)_6_(OH)_2_, is the reference mineral in the studies of synthetic and biological apatites. Generally, apatites are non-stoichiometric, although they keep the same crystal space group *P*6_3_/*m*. They are prone to various ionic substitutions in the crystal lattice, which substantially affect their physicochemical properties and biological behavior.

The HA mineral of mammal bone, dentin, and dental cementum is nanocrystalline. It is formed of prolate platelet crystals, which are 30–50 nm long, 15–30 nm wide, and 2–10 nm thick (Dorozhkin 2009, 2010). Because of the nanosize of those crystallites, their surface and interior are considerably interrelated, which circumstance gives the material unique properties. Such biological HA contains 3–9 wt % of CO_3_^2−^ ions, preferably substituted for PO_4_^3−^ ions in so-called B-sites (Aoki 1991; Dorozhkin 2009, 2010; Eichert et al. 2009; Elliott 1994; LeGeros 1991; Olszta et al. 2007). The nanocrystals are covered by a hydrated layer, which accommodates ionic non-apatitic environments (Bertinetti et al. 2007; Bertinetti et al. 2008; Cazalbou et al. 2004; Jäger et al. 2006a, b; Kaflak and Kolodziejski 2008; Ramirez et al. 2009; Rey et al. 2007; Rey et al. 2009; Sakhno et al. 2010; Wilson et al. 2005, 2006). This layer is 1–2 nm deep and encompasses 40–55 % of phosphorus present in the material. Some researchers assume this region to be structured, while others describe it as amorphous. The hydrated layer participates in an ionic exchange and serves as a functional interface between mineral and proteinaceous compartments of hard tissues.

In the HA crystal lattice there are 0.30–0.35 nm wide channels, running along the crystal *c*-axis. They can be occupied by structural hydroxyl groups (OH^−^ ions) (Aoki 1991; Elliott 1994; LeGeros 1991), other small ions as F^−^, Cl^−^, CO_3_^2−^ (the latter species called A-type carbonates) (Aoki 1991; Elliott 1994; LeGeros 1991) and structural water (Ivanova et al. 2001; Mason et al. 2009; Nordström and Karlsson 1990; Wilson et al. 2005, 2006; Yoder et al. 2012a, b). In stoichiometric HA, those channels contain only the hydroxyl groups, organized in the ···O–H O–H O–H O–H··· columns. The consecutive hydroxyls are too distant from each other to form hydrogen bonds (the O–O distance of 3.44 Å). Therefore, their proton NMR peak recorded under magic-angle spinning (MAS) appears at 0.0 ppm (Kolodziejski 2005). Carbonated HA of bone mineral contains only ca. 21 % of the structural hydroxyl groups, normally present in the stoichiometric HA (Cho et al. 2003). For dental enamel, dentin, and dental cementum, the respective values are 73, 18, and 18 % (Kolmas and Kolodziejski 2007). Pasteris et al. (2004) and Wopenka and Pasteris (2005) proposed an interesting explanation of those OH^−^ deficiencies in apatites. They postulated that the smaller the crystallite size, the greater is atomic disorder within crystal unit cells and the less energetically favorable it is for apatite to incorporate OH^−^ into its channel sites.

In this work, our primary objective was to verify experimentally that hypothesis using a series of nanocrystalline apatites with decreasing mean crystal sizes, prepared by dry milling of the same parent material. As far as we know, a relationship between structural hydroxyl groups and surface hydrated layer has been studied for the first time. Mechanochemical synthesis, activation, and modification of HA have already been investigated (Abdel-Aal et al. 2008; Bignon et al. 2002; Chaikina and Aman 2005; Isobe et al. 2002; Mohammadi Zahrani and Fathi 2009; Nakano et al. 2001; Nasiri-Tabrizi et al. 2009; Nilpairach 1997; Ruksudjarit et al. 2008). However, solid-state NMR has been used only once in mechanochemistry of apatites, that is, to characterize HA prepared by a wet-mechanochemical reaction (Isobe et al. 2002). We employed ^1^H and ^31^P MAS NMR to study dry-milled samples as a function of the milling time and crystal size. The proton peaks at 0.0 and 5.4 ppm were applied to quantify structural hydroxyl groups and water, respectively. Cross-polarization (CP) from the hydroxyl and water protons was used to analyze a complex ^31^P MAS NMR line at 3.1 ppm. Components of that line allowed us to monitor the crystal interior and the crystal surface. Dependencies of various physicochemical properties on the crystal size have been also examined and discussed.

## Materials and methods

### Samples and their characterization

The foremost parent hydroxyapatite designated HA0 was purchased from Berkeley Advanced Biomaterials, Inc. (BABI-HAP-SP; agglomerated sphere-like dry powder, average particle size of ca. 5 μm and the Ca/P ratio of 1.67, as declared by the supplier). According to our later examination using transmission electron microscopy (TEM), the particles were formed of ca. 100 nm crystals. This material lost ca. 0.19 and 0.32 wt % in the 25–200 and 200–550 °C temperature ranges, respectively (TGA using SDT Q600 V20.9 Build 20 of TA Instruments; measured in argon, 10 mg samples, heating rate of 5°/min, no thermal pre-treatment before performing the TGA analyses). Those quantities correspond to adsorbed water and structural water, respectively (Yoder et al. 2012a, b), giving HA0 the total water content of 0.51 wt %.

HA0 was subjected to dry milling in a vibrational ball mill (Testchem; LMW-S) for a specified period of time *t* in order to obtain apatites with various mean crystal sizes. Accordingly, the milled samples have been designated HA*t*, where *t* is given in hours. The milling was done in tungsten carbide vessels using tungsten carbide 12 mm balls. Each milling session to produce particular HA*t* started from 1 g of HA0. After every hour of processing, the procedure was interrupted for 1.5 h in order to cool down the device (no breaks for HA0.25, HA0.5, and HA1).

It has been checked using powder X-ray diffraction (PXRD; Bruker D8 Discover), that the HA*t* samples were homogenous and contained only apatite. As well, their ^1^H and ^31^P MAS NMR spectra were typical of HA (vide infra). PXRD was also used to determine a crystallinity parameter according to Landi et al. (2000). As the milling operation was done in the air atmosphere (external temperature at 298 K, humidity of ca. 40 %), the material was allowed to absorb ambient CO_2_ and to undergo CO_3_^2−^ substitutions for PO_4_^3−^ groups (type-B carbonates) or for structural OH^−^ groups (type-A carbonates). The contents of the A and B carbonates were estimated using the infrared method of Sønju Clasen and Ruyter (1997) and (Kaflak et al. 2011). Spectroscopic FT-IR studies were carried out at 298 K on a Perkin Elmer Spectrum 1000 spectrometer. The absorption spectra were acquired with 2 cm^−1^ spectral resolution from KBr pellets using a mercury cadmium telluride (MCT) detector and 50 scans. They were processed using the GRAMS/AI 8.0 software (Thermo Scientific, 2006). The processing included baseline correction, offset correction, and peak fittings.

Apatite crystals (non-sintered) were observed using TEM (JEOL JEM 1400 equipped with the EDX accessory). For this purpose, a drop of a sample suspension in ethanol (without prior ultrasonication) was placed on a Ni grid covered with a Formvar film, allowed to dry and analyzed under the accelerating voltage of 80 kV. Averaged crystal sizes were calculated from at least 310 randomly selected 2D crystal images using the STATISTICA 64 software (Version 10, StatSoft, Inc. 2011). The electron diffraction studies were done on a JEOL TEM microscope (JEM 2000EX). The diffraction images were recorded on a photographic film and then converted to digital images with a NIKON LS-8000 scanner. The sample suspension in methanol was ultrasonicated over 45 min and then a drop of this suspension was placed on a Cu grid covered with a carbon film, allowed to dry and analyzed using an electron beam with energy of 200 keV.

The HA0 crystals were observed in the 3D mode by electron tomography using a JEM 1400 (Jeol Co.,) microscope at 80 kV with a tilt-rotate tomographic holder and a high-resolution digital camera (CCD MORADA; Olympus Soft Imaging Solutions, Germany). The images were taken at 60,000 magnification from 55.4° to −55°, in steps of 0.82°. The series of 134 images with the defined common axis of rotation was exported in TIFF (multi-page). The latter format was then converted into AVI using ImageJ software (National Institutes of Health, Bethesda, MD, USA).

Agglomerates of apatite crystals were observed using NovaNano SEM 450 electron microscope (FEI), equipped with the EDAX accessory (Apex/Genesis XM 2 with an Apollo XL SDD detector). The apatite powder was fixed to an adhesive conducting tape and then analyzed under the accelerating voltage of 5 kV.

The physicochemical characteristics of the studied samples are presented in Table 1. Representative PXRD diffractograms and FT-IR spectra are shown in Online Resource 1.

**Table 1:** Physicochemical characteristics of the HA0–HA36 samples

Sample	Mean crystal size^a^ (nm)		Crystallinity index^b^	TGA weight loss^c^ (wt%)	B carbonates^d^ (wt%)	Structural OH content^e^	Relative water content^e^
	Length	Width					
HA0	129	95	94	0.51	0.49	86.4	100
HA0.25	97	71	ND	ND	0.46	86.8	99
HA0.5	81	61	ND	ND	0.58	85.6	122
HA1	82	58	ND	ND	0.54	85.6	217
HA2	85	62	ND	ND	0.57	80.8	246
HA3	56	44	84	1.75	0.61	78.3	341
HA4	55	40	ND	ND	0.58	75.8	311
HA5	56	44	ND	ND	0.63	67.3	365
HA6	63	44	75	2.65	0.59	66.5	329
HA12	42	32	65	3.66	0.69	60.6	487
HA24	35	26	58	4.50	0.96	50.8	616
HA36	23	20	61	3.78	0.90	53.2	957

^a^TEM (see Materials and methods and Online Resource 1)

^b^From PXRD (Landi et al. 2000)

^c^In the 25–550 °C temperature range

^d^From IR (Kaflak et al. 2011; Sønju Clasen and Ruyter 1997)

^e^From proton MAS NMR

### NMR experiments

High-resolution solid-state ^1^H and ^31^P NMR spectra (Bruker Avance 400WB; 400 MHz for ^1^H; 160 MHz for ^31^P) were acquired with MAS at 12 kHz for ^1^H, and 7 and 3.5 kHz for ^31^P. The rotors were spun by dry air at 298 K. The conventional π/2 pulse-acquire (Bloch-decay, BD) and CP spectra were measured using a Bruker 4 mm probe. The ^1^H BD/MAS NMR experiments were done with a π/2 ^1^H pulse of 2.9 μs, 32 scans and a repetition time of 30 s. The proton background of the probe was carefully subtracted (Chen et al. 2004). The ^31^P CP/MAS NMR experiments were done with a π/2 ^1^H pulse of 2.65 μs, a contact time of 2 ms, 64 scans and a repetition time of 5 s.

The ^31^P CP/MAS NMR signal was also recorded with a specific selection of water or structural OH groups as the proton polarization source. This was done by appropriate preparation of the proton reservoirs before CP. Water was well selected by a presaturation comb of proton pulses followed by a properly adjusted relaxation delay, which pulse chip preceded the normal CP pulse sequence on the proton channel. That introductory segment of the modified CP pulse sequence worked similarly to the conventional saturation recovery experiment performed on proton spins and took advantage of shorter *T*_1_ of the water protons comparing to that of the intracrystalline OH protons. Structural OH groups were selected by an extra proton lock of 15 ms, which was introduced before the contact pulse on the proton channel (Kaflak and Kolodziejski 2008; Kolodziejski 2005). Such experiment was capable of eliminating the magnetization of the water protons, since their *T*_1*ρ*_ was much shorter than that of the structural OH protons.

Relative contents of structural OH groups and water were determined by fitting the 0.0 and 5.4 ppm proton lines, respectively, followed by comparing their areas to those of appropriate standards. The calculations involved sample weights. The measurements for the studied samples and standards were done using the same BD pulse sequence, MAS rate, and NMR parameters. The probe tuning required only a minor correction for the next apatite sample. As the OH standard, we used the HA800 sample from the former work (Kolmas and Kolodziejski 2007) (calcined at 800 °C; 94 ± 1 % of OH by reference to stoichiometric HA). The relative water content was determined by reference to the parent apatite HA0.

The peak fittings were done using the NutsPro (Acorn NMR 2007) and ACD SpecManager (Version 10.08, Advanced Chemistry Development, Inc., 2007) computer programs. Figures were prepared using the KaleidaGraph computer program (Version 3.5 for PC, Synergy Software, 2000).

## Results and discussion

### General characteristics of the milled apatites

**Fig. 1:**
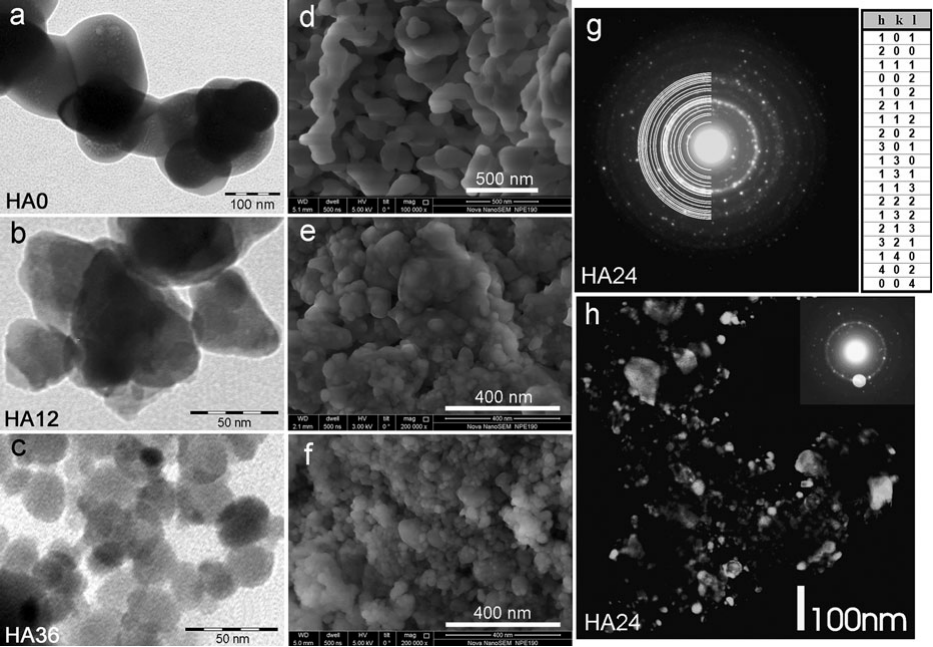
The morphology and crystallinity of the studied apatites: **a**–**c** representative TEM micrographs; **d**–**f** representative SEM micrographs; **g** electron diffractogram of HA24 (*left*) with Miller indices (*right*) assigned to the diffraction rings and listed from the pattern center; **h** a dark field image of HA24 formed from the strongest reflections: (2 1 1) and (1 1 2) (see the *upper-right inset*). The parameters of the hexagonal unit cell of HA24, determined from the electron diffraction: *a* = 0.9424(4) nm, *c* = 0.6879(4) nm

On the bright field TEM micrographs, 2D images of the HA0–HA36 crystals had prolate plate-like shapes and nanodimensions (Fig. 1a–c). Those dimensions were determined for single, well-separated crystals or well-seen crystals belonging to small grains, selected from many TEM micrographs to satisfy random sampling requirement. In manual treatment (Higgins 2000), a selected 2D crystal image was analyzed to find its maximum length and orthogonal width with a measure IT program (Version 5.1, Build 2067) of Olympus Soft Imaging Solutions GmbH. Then, those characteristics were employed as measures of the true crystal length and width, respectively. The validity of this procedure will be discussed later. The crystals had strong tendency to agglomeration, as shown using SEM (Fig. 1d–f). According to the electron diffraction (Fig. 1g), the studied samples were polycrystalline and contained apatite with a hexagonal crystal structure. Moreover, no admixed crystalline phases were found using PXRD (Online Resource 1). The dark field TEM images demonstrated variation of sizes and shapes of the material nanoparticles (Fig. 1h).

**Fig. 2:**
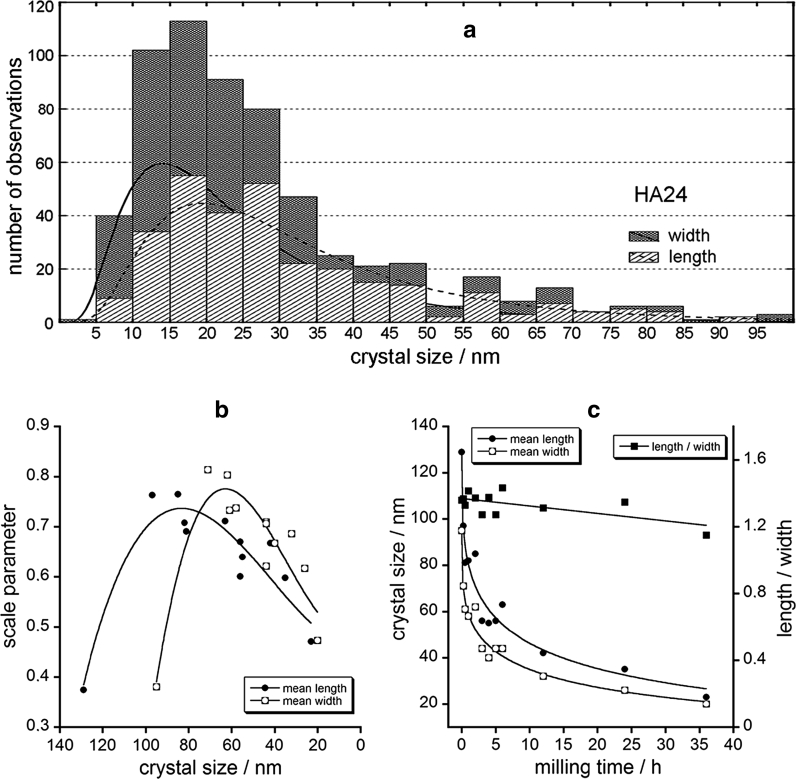
The crystal size distributions and mean crystal dimensions for the studied apatites: **a** stacked lognormal distributions of crystal lengths and widths in HA24; **b** the dependence of the scale parameter of the lognormal distribution on the mean crystal dimensions of various samples; **c** the dependence of the mean crystal dimensions and the length/width aspect ratio on the milling time. The curves in the parts **b** and **c** are only guides for eyes

We were also interested in true 3D habits of the apatite crystals. For HA0 (the largest crystals), it was feasible to acquire electron tomography movie (Online Resource 2). This revealed stubby rod-shaped crystals. The chunky rods with rounded terminations were also visible on the SEM micrographs of the HA0–HA36 samples (Fig. 1d–f). Thus, the similar 3D shape was maintained in the series of our samples, which circumstance rendered the length-to-width aspect ratio almost invariant to the milling time (1.33 ± 0.02, cf. Table 1; Fig. 2). The latter effect is also consistent with the fractal theory of material fragmentation by crushing, grinding, blasting, and so on (Turcotte 1986). In our case, apatite particles obtained by dry milling probably follow a self-similar (fractal) size distribution (Glazner and Mills 2012).

During the analysis of the TEM micrographs one is confronted with the stereological problem of using 2D projection (section) data to construct 3D crystal size distributions (Higgins 2000; Royet 1991). For the stereological data treatment, both the cut-section effect and the intersection-probability effect have to be operative (Higgins 2000). Those requirements are not fulfilled in our work. In fact, we found no indications in the electron diffractograms of a specific crystal alignment, e.g., no oblique texture electron diffraction pattern. However, our crystal selection procedure for the TEM analysis was focused on individual crystals, which should rather have tendency to deposit their rods parallel to the Formvar film. Therefore, we infer that the crystal length and size values obtained from the 2D images are sound measures of the corresponding 3D structural parameters. Anyway, we had to rely on those values, because there are no dependable stereological procedures for rod-shaped crystals to obtain true 3D structural parameters from their 2D counterparts. Furthermore, for a preferable alignment the conventional stereological data processing fails.

For each sample, the distributions of the TEM-determined crystal length and width were lognormal (Fig. 2a). In the lognormal distribution, the scale parameter *σ* is the standard deviation of the data set after the normal-logarithmic transformation. The lognormal distribution is skewed right, and the skewness increases as the value of *σ* increases. The skewness of our distributions of crystal dimensions steeply increased at the beginning of milling, reaching maximum *σ* for HA0.25–HA0.5, and then decreased with the decrease of the crystal size (Fig. 2b). The mean crystal dimensions decreased on milling (Fig. 2c), especially during first 5 h of grinding. After 36 h milling, the crystal size decreased from ca. 100 nm to ca. 20 nm, while the crystallinity assessed by the PXRD index (2000) went down from 94 to ca. 60 % (Table 1). The BET surface area increased from 5 to 46 m^2^ g^−1^. Our parent apatite HA0 contained ca. 0.5 wt % of B carbonates. On milling, their concentration increased to ca. 1.0 wt % for HA24 and HA36, as estimated from the 1,415 cm^−1^ IR band (Kaflak et al. 2011; Sønju Clasen and Ruyter 1997). For all studied samples, the content of A carbonates was immaterial (the A/B carbonate ratio below 0.05), as estimated from the 1,544 cm^−1^ IR band (Kaflak et al. 2011; Sønju Clasen and Ruyter 1997). The concentrations of structural OH groups and water decreased and increased with the milling time, respectively (Table 1), and those findings will be of our particular concern in the following discussion.

### Interpretation of the NMR spectra

**Fig. 3:**
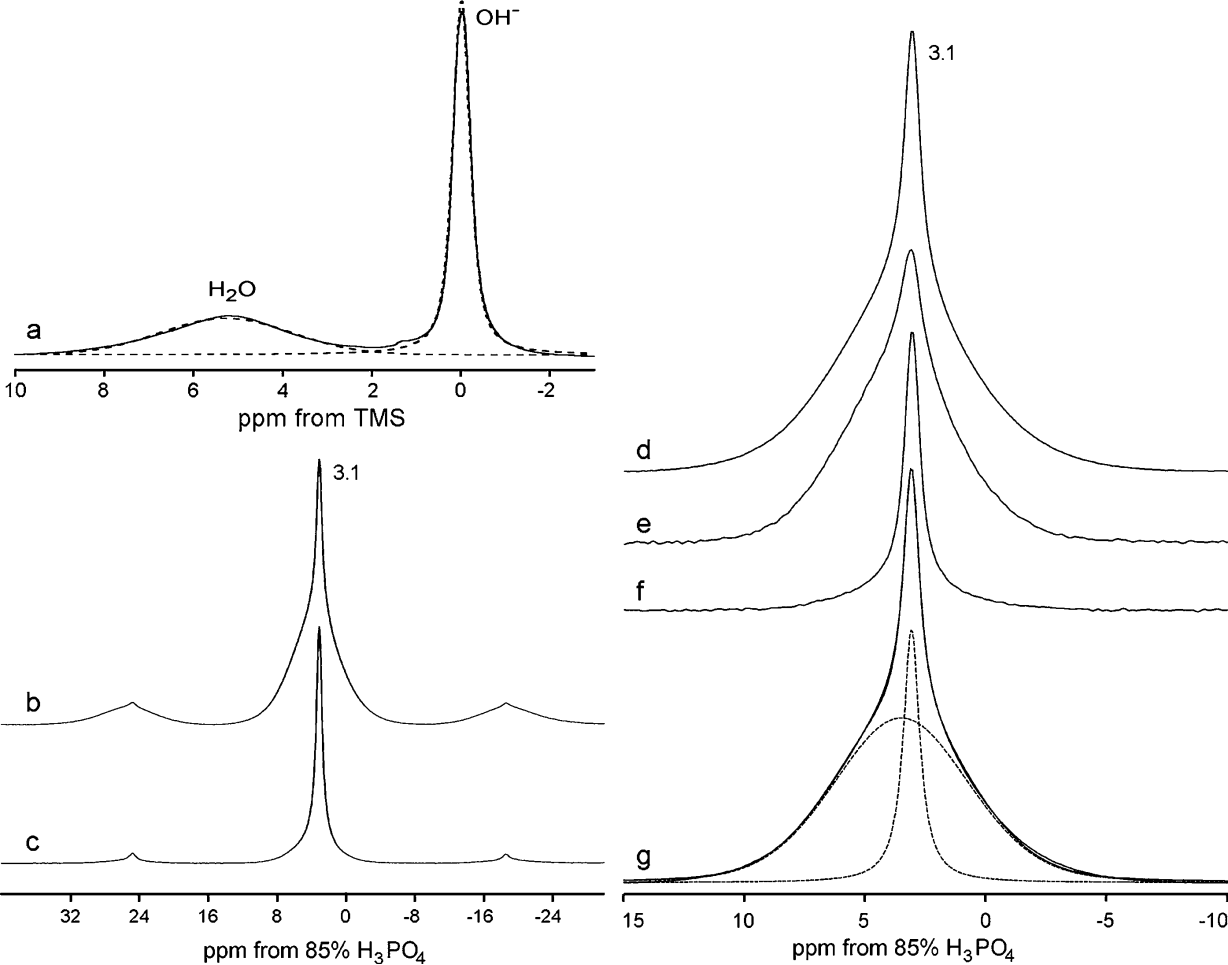
The exemplary solid-state NMR spectra of the apatite powders, shown for HA24: (*a*) ^1^H NMR under MAS at 12 kHz; (*b*)–(*g*) ^31^P NMR spectra under MAS at 3.5 kHz. The graphs (*b*) and (*c*) present a comparison of the CP and BD spectra, respectively, including first spinning sidebands. Next three spectra were recorded under various preparations of the proton reservoir before CP (centerbands only shown): (*d*) a regular signal; (*e*) presaturation of protons of structural hydroxyl groups; (*f*) an extra 15 ms proton lock before CP (magnetization of water has been eliminated). In the graph (*g*), the regular ^31^P CP line was deconvoluted into two components: a narrow one from the crystal interior (close to the Lorentzian shape) and a broad one from the hydrated layer (Gaussian). The CP spectra were acquired with the 2 ms contact time

The proton MAS NMR spectra of the milled apatites (Fig. 3a) contain two signals at 0.0 and 5.4 ppm from structural hydroxyl groups and water, respectively (Kolodziejski 2005). Water resonating at 5.4 ppm has diverse nature. It can be adsorbed and structural. The corresponding ^1^H chemical shift is characteristic of hydrogen bonded water. It follows that molecules of the structural water, residing in the *c*-axis channels, form hydrogen bonds among themselves and with hydroxide ions, as it was reported by Wilson et al. (2006). Molecules of the adsorbed water are dissimilar in terms of molecular interactions. They can be coordinated to surface Ca^2+^-sites or can form hydrogen bonds with surface P–OH and P=O sites, and all of them can participate in hydrogen bonding with neighboring water molecules (Bertinetti et al. 2007; Bolis et al. 2012; Ishikawa et al. 1989; Wilson et al. 2006). The surface species are involved in a proton exchange via hydrogen bonds (Kaflak-Hachulska et al. 2003; Kaflak and Kolodziejski 2007, 2011; Kolodziejski 2005), which process controls the shape and chemical shift of the proton line at 5.4 ppm.

The ^31^P MAS NMR spectra of various samples all show a composite signal with a peak position at ca. 3.1 ppm (Fig. 3b, c). That chemical shift is characteristic of apatite (Kolodziejski 2005). A visual inspection of the CP (Fig. 3b) and BD (Fig. 3c) spectra assures that this composite signal comprises at least two overlapped components, probably narrow one at the top of a broad massive line. The latter line is clearly exposed by CP and gives predominant contribution to spinning sidebands.

The composition of the ^31^P NMR signal (Fig. 3d) was simply and reliably determined using CP with an appropriate selection of the polarization source. When polarization was transferred from protons of water, the NMR experiment produced predominantly the broad ^31^P component (Fig. 3e). By contrast, CP from protons of structural OH groups generated mostly the narrow ^31^P component. Those discriminating experiments could not give absolutely neat components because of proton spin diffusion between both proton environments. Therefore, we finally evaluated the ^31^P signal components using peak fittings (Fig. 3g). The mean chemical shifts of the narrow and broad components were found to be 3.10 ± 0.01 and 3.65 ± 0.06 ppm, respectively.

The above-mentioned discriminating experiments helped us to assign the broad component to P-sites residing in a water-rich environment and the narrow component to P-sites located close to structural hydroxyl groups. The water-rich environment must be primarily associated with a surface hydrated layer of apatite crystals and possibly can be related to some disordered intracrystalline regions containing water molecules (structural water). The environment of the structural hydroxyl groups can be undoubtedly identified with the crystal interior. The almost pure Gaussian shape of the broad component is indicative of a disorder present in the surface hydrated layer. As concerns the narrow component, it was clearly changing its shape on milling (not shown) from mixed Lorentzian-Gaussian to Gaussian. This NMR effect was probably caused by some influence of the increasing hydrated layer on the crystal interior. We believe that the progressive hydration imposes some structural disorder on the crystal lattice of apatite nanocrystals (vide infra).

**Fig. 4:**
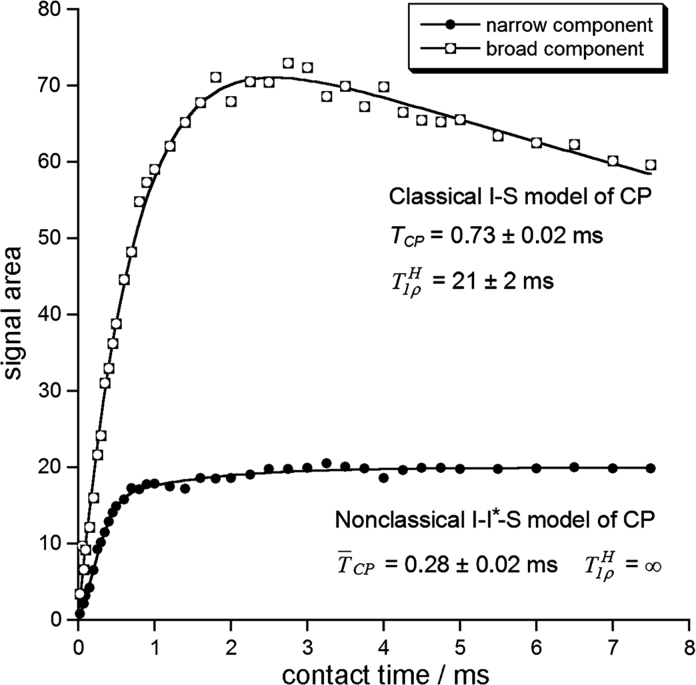
The dependence of the components of the ^31^P CP/MAS NMR signal on the contact time (HA24, MAS at 3.5 kHz). The fitted curves correspond to the nonclassical and classical CP kinetics (Kaflak and Kolodziejski 2008; Kolodziejski and Klinowski 2002; Kolodziejski 2005) for the narrow and broad components, respectively

The above assignments of the narrow and broad ^31^P MAS NMR components are in agreement with the former NMR studies of synthetic and biological apatites carried out in our group (Kaflak and Kolodziejski 2008; Kolodziejski 2005). Those studies explained CP kinetics in synthetic, carbonated HA and in bone HA using together two different CP models (Kolodziejski and Klinowski 2002). In the present work, we also found that CP from water, producing here the broad component, conforms to the classical CP model and that CP from structural hydroxyl groups, producing here the narrow component, complies with the nonclassical CP model (Fig. 4). As in the previous studies (Kaflak and Kolodziejski 2008; Kolodziejski 2005), the proton relaxation times in the rotating frame $T_{ 1\rho }^{H}$ determined from variable-contact time experiments, were short (21 ms for HA24) and indefinitely long for the classical and nonclassical CP kinetics, respectively.

Hereafter, when discussing the broad component, we shall use CP results for the optimum contact time of 2 ms (Fig. 4). Either BD or CP results will be presented to emphasize quantitative or accuracy aspects, respectively. For the same reason, the results obtained under different MAS rates will be selectively considered in the subsequent figures. For MAS at 3.5 kHz, the ^31^P NMR lines were broader, so their linewidths were determined more accurately than for MAS at 7.0 kHz. Conversely, the signal areas were less accurate because spinning sidebands had to be included in the signal deconvolutions. But anyway, the complementary remaining results are presented as Online Resource 1.

The interpretation of the ^31^P MAS NMR spectra requires adequate caution. We submit that the broad component cannot be from ordinary amorphous phosphate (ACP), because ACP gives different ^1^H and ^31^P MAS NMR spectra than our milled apatites (Online Resource 1). Powder X-ray diffractograms and FT-IR spectra of the milled samples are typical of fairly crystalline apatites (Online Resource 1). ACP is known to form Posner’s clusters (Boskey 1997), which give broad diffraction halos and produce a ν_1_-phosphate IR band at about 950 cm^−1^ (Combes and Rey 2010). No such features were found for our samples, even though HA24 and HA36 contained about 30 % of P in the hydrated layer (vide infra). Nevertheless, the hydrated layer of our milled apatites can still have a different amorphous arrangement to that involving Posner’s clusters. There are also other assignments in the literature for extra peaks accompanying the main ^31^P MAS NMR resonance of apatite. Jarlbring et al. (2006) assigned two weaker shoulder peaks at 5.4 and 0.8 ppm to unprotonated ≡PO_*x*_ and protonated ≡PO_*x*_H surface sites in fluoroapatites (≡ designates a chemical bond with the HA surface). Wu et al. (1994) found in bone apatite a signal at −0.4 ppm from unique HPO_4_^2−^ ions. However, our broad component appeared at ca. 3.65 ppm and the aforementioned resonances were absent.

Overall, the proton and ^31^P MAS NMR peaks from milled apatites have been unequivocally assigned. The proton NMR signals at 0.0 and 5.4 ppm from structural hydroxyl groups and water, respectively, can be used to calculate concentrations of those chemical species. In addition, the narrow (3.10 ppm) and broad (3.65 ppm) components of the ^31^P MAS NMR signal can be employed to monitor environments of structural OH groups (crystal interior) and of water (crystal surface for adsorbed H_2_O and crystal interior for structural H_2_O), respectively.

### Content and location of water

**Fig. 5:**
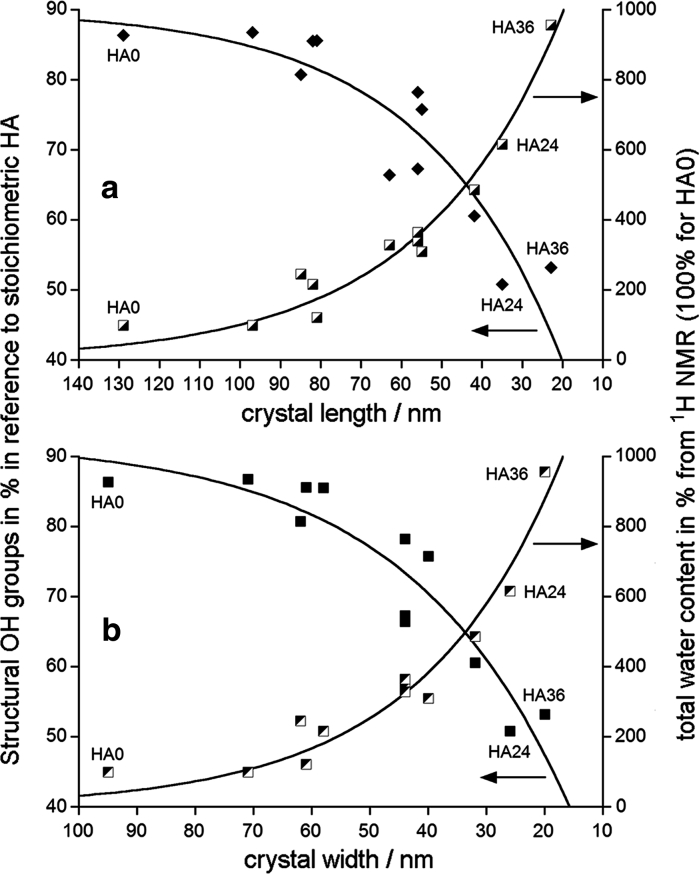
The dependence of the concentrations of structural OH groups (in % in reference to stoichiometric HA) and the total water content (in %, 100 % for HA0) on the mean crystal dimensions: **a** on the crystal length; **b** on the crystal width. The OH and H_2_O concentrations were determined using proton MAS NMR. The exponential curves are only guides for eyes

The total water content, determined from ^1^H MAS NMR, increased during milling by one order of magnitude (Table 1). This was caused by a humidity uptake from the surroundings (external temperature at 298 K, humidity of ca. 40 %). The total water content increased with the decreasing mean crystal length and width (Fig. 5) because of an increasing surface area of crystallites. The corresponding results from TGA indicate an eight- to ninefold increase of the total water content (from 0.5 to 3.8–4.5 wt %, Table 1), as estimated from the weight loss in the 25–550 °C temperature range (Yoder et al. 2012a, b). The ^1^H MAS NMR and TGA results show significant mutual linear correlation (*R* = 0.9724, *p* = 0.0055; see Online Resource 1). Hence, they are in good agreement as the total water content is concerned.

**Fig. 6:**
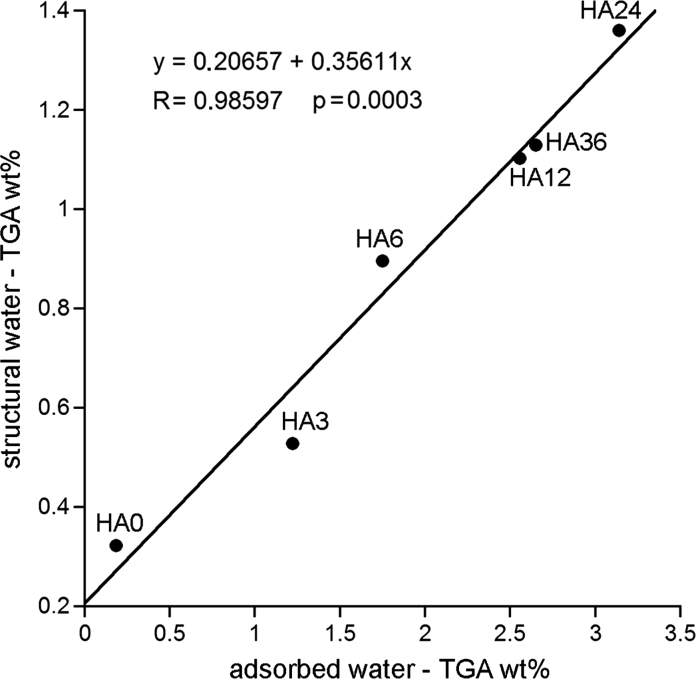
The relationship between the structural and adsorbed water contents in the milled apatites. The water contents were determined using TGA from the weight losses in the 200–550 and 25–200 temperature ranges (°C), respectively

It has recently been proposed to estimate adsorbed and structural water from TGA weight losses in the 25–200 and 200–550 °C temperature ranges, respectively (Yoder et al. 2012a, b). Following this interpretation we found using TGA that the contents of the adsorbed and structural water increased on milling and that their ratio increased from 0.6 for HA0 to 2.3 for HA12–HA36 (Fig. 6). Moreover, those concentrations demonstrated a significant positive linear correlation, so that the adsorbed water could be somehow converted to the structural water. A simplest explanation would be that molecules of the adsorbed water are capable of moving from the surface of apatite into its crystal lattice.

Indeed, water molecules from the crystal surface can enter intracrystalline *c*-axis channels used by structural hydroxide ions (Kaflak and Kolodziejski 2008, 2011; Wilson et al. 2006; Yoder et al. 2012a). In the former works from our group (Kaflak-Hachulska 2000; Kaflak and Kolodziejski 2008) it has been postulated that there is probably a proton exchange involving hydroxide ions and water in those channels. Finally, Yoder et al. (2012a) proposed that such process might aid water to get into the crystal lattice channels and that this mechanism could be especially effective at higher temperatures (Yoder et al. 2012a). Since high temperatures are attainable during milling, we suppose that the proton transfer mechanism was in operation for our samples. It was probably supported by physical diffusion of water molecules to fill voids in the *c*-axis channels (Kaflak and Kolodziejski 2008, 2011; Wilson et al. 2006).

**Fig. 7:**
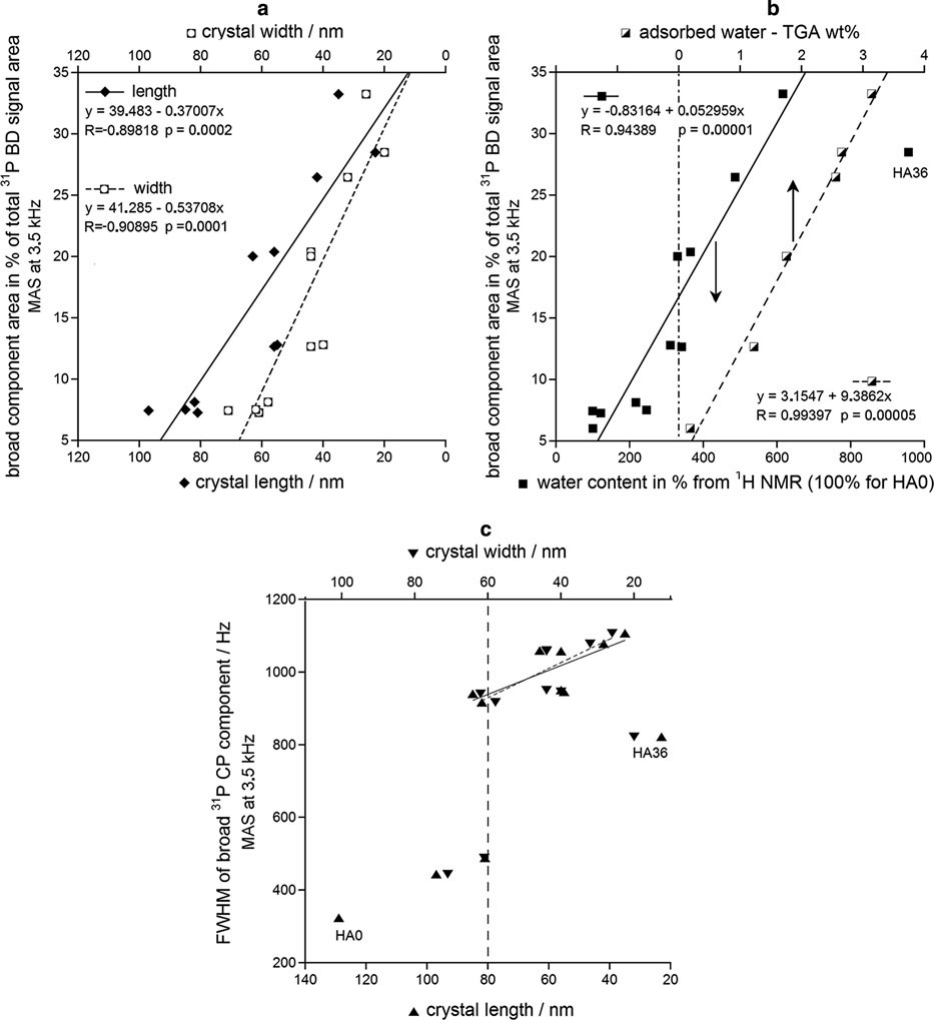
The parameters of the broad component of the ^31^P MAS NMR signal plotted against various characteristics of the milled apatites: **a** contribution to the BD spectrum (area in %) versus mean crystal dimensions; **b** contribution to the BD spectrum (area in %) versus total and adsorbed water contents; **c** FWHM from the CP experiment versus mean crystal dimensions. The experiments were done with MAS at 3.5 kHz, first spinning sidebands have been included in the deconvolutions and calculations, CP was performed with the contact time of 2 ms. The total and adsorbed water contents were determined using proton MAS NMR and TGA, respectively

Environments of the adsorbed and structural water can be monitored using the broad ^31^P MAS NMR component. According to our BD studies (Fig. 7a), the broad component corresponded to ca. 30 % of apatite phosphorus for the lowest crystal sizes (HA24 and HA36). It is reminiscent of our CP study of synthetic carbonatoapatite and bone mineral (Kaflak and Kolodziejski 2008), which reported that the surface hydrated layer contained ca. 40 % of apatite phosphorus. In the present work, the broad component area shows significant negative linear correlation with the crystal length and width (Fig. 7a) because the environments of water relatively increase with the decreasing crystal size. Of course, the environments of water increase with the increasing total water content (^1^H NMR) and with the increasing concentration of the adsorbed water (TGA). Those dependencies are confirmed by respective significant linear correlations in Fig. 7b. The relationship with the adsorbed water was chosen because this kind of water was generally more abundant in our samples (Fig. 6).

A rise in the linewidth of the broad ^31^P MAS NMR component can be indicative of a higher disorder in the water environments. Unsuccessfully, the FWHM results for the broad components of the HA0–HA0.5 samples were not sufficiently precise. Therefore, we did not attempt to fit the points in Fig. 7c with a typical sigmoidal activation function (see also Online Resource 1). However, we are convinced of a sharp increase in FWHM for the crystal length of 80 nm and the crystal width of 60 nm. We believe that for those crystal dimensions the surface hydrated layer began to take form typical of nanocrystalline apatites. The reported crystal dimensions of the hydration transition are probably specific to the milling conditions.

### Structural hydroxyl groups

The content of the structural hydroxyl groups (hydroxide ions in the *c*-axis channels) decreased on milling by ca. 40 % in reference to stoichiometric apatite (Fig. 5). The small increase in B carbonates (Table 1) cannot be responsible for such effect. In terms of the crystal size this decrease occurred in the 130–30 nm range of the crystal length and in the 95–20 nm range of the crystal width. Thus it was confirmed that the content of the structural OH groups in nanocrystalline apatites decreases with the decreasing crystal size (Pasteris et al. 2004; Wopenka and Pasteris 2005). We submit that the content of the structural OH groups did not go down in our samples to the level of ca. 20 %, typical of nanocrystalline biological apatite of bone, dentin, and dental cementum. Perhaps, the content of the structural OH groups in calcified tissues is also biologically controlled during biomineralization.

**Fig. 8:**
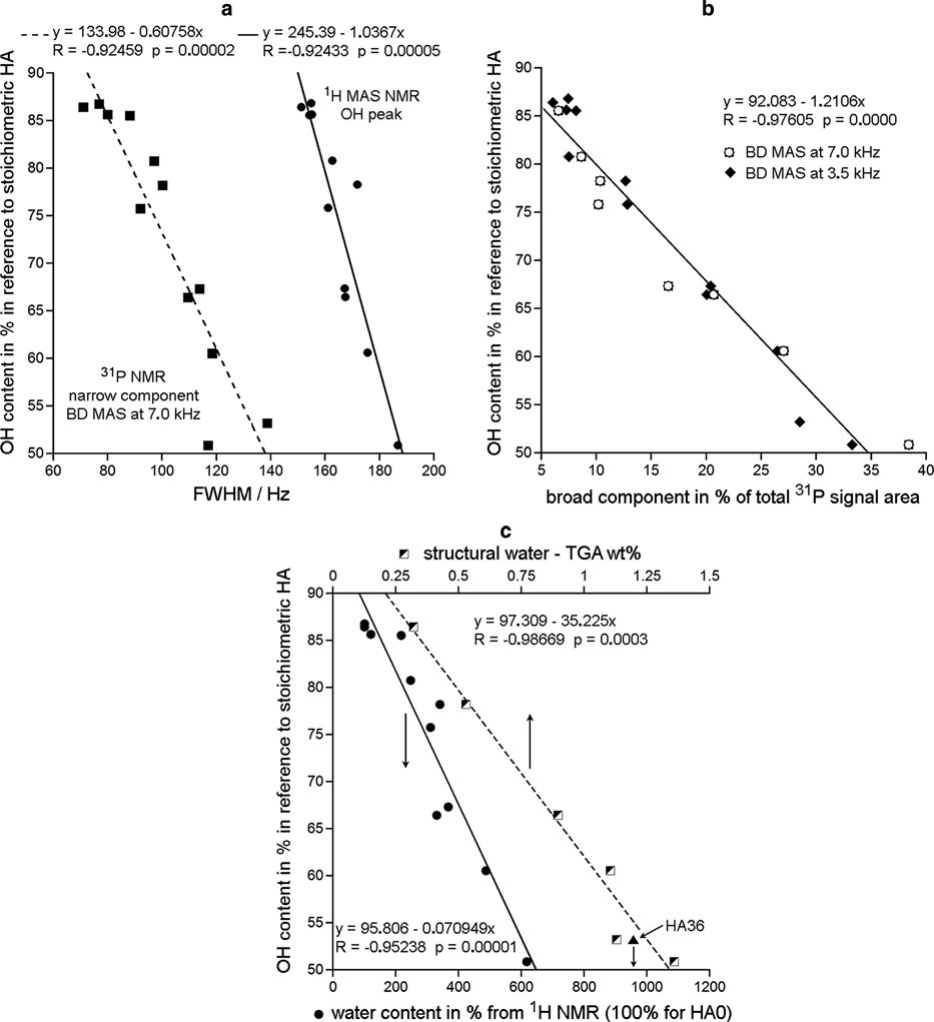
The content of structural hydroxyl groups (in % in reference to stoichiometric HA) plotted against following characteristics of the milled apatites: **a** FWHM of the narrow ^31^P BD/MAS NMR component (MAS at 7 kHz) and FWHM of the ^1^H MAS NMR hydroxyl peak (MAS at 12 kHz); **b** contribution of the broad component in % to the total ^31^P BD/MAS NMR signal area; **c** total and structural water contents from proton MAS NMR and TGA, respectively (see text). In the graph **b** points for MAS at 7.0 and 3.5 kHz have been treated together, for the slower MAS rate first spinning sidebands were included in the deconvolutions and calculations

In order to find a reason of the OH deficiency for smaller crystallites, we examined relationship between the structural OH content and appropriate MAS NMR linewidths (Fig. 8a). As before it has been assumed that a broader line indicates a higher disorder. The FWHM value of the proton peak at 0.0 ppm was selected to probe the state of the structural hydroxyl groups. The FWHM value of the narrow ^31^P BD component at 3.1 ppm was selected to probe the state of the apatite crystal lattice around the *c*-axis channels containing hydroxide ions of interest and structural water. We found that the structural OH content decreased with the increase of both FWHM variables showing significant linear correlations (Fig. 8a). Those correlations can be interpreted as the decrease of the structural OH content with the growing disorder within and around the *c*-axis channels, according to the idea of Pasteris et al. (2004) and Wopenka and Pasteris (2005).

The reason of this disorder has been revealed by considering the dependence of the structural OH content on the amount of the hydrated apatite environments, monitored using the broad ^31^P component area (Fig. 8b), and on the total water content (^1^H NMR) and the structural water content (TGA) (Fig. 8c). In all those cases we found significant negative linear correlations. They indicate that the studied disorder, diminishing the structural OH concentration, is probably caused by an uptake of the structural water into the *c*-axis apatite channels. Such explanation has already been given in our former work (Kaflak and Kolodziejski 2011). That water uptake should efficiently decrease the structural OH content particularly in the region underneath the apatite crystal surface. It is in accordance with the conclusion of Wilson et al. (2005) that “hydroxide ion is present in a limited portion of the crystal, most likely the internal region of the bone mineral crystallites and not near the disordered surface regions”.

### Special case of sample HA36

This sample had the smallest crystals (Table 1). We admit that the points for HA36 in several linear regression graphs were typical outliers. Comparing to HA24, HA36 contained less structural water (Fig. 6), less disordered water environments (Fig. 7c), more total water (Fig. 8c, cf. NMR estimation in Table 1) and slightly more structural OH groups (Table 1). It follows that HA36 had larger surface hydrated layer and lower concentration of structural water than expected from the milling time. We found that HA36 gave different EPR spectrum, generated using high energy γ-radiation (extra O^−^ radicals located on the crystal surface, not present in other samples) (Sadło et al. 2012). As yet we have no idea why this sample did not match the whole series. We speculate that the reason had to be related to the exhausting milling.

## Conclusions

Using the series of HA0-HA36 samples we carried out model ^1^H and ^31^P MAS NMR, TEM, and TGA studies of structural hydroxyl groups and water in dry-milled (up to 36 h) apatites with various crystal sizes. The structural hydroxyl groups and water were monitored in the 130–30 nm range of the crystal length and in the 95–20 nm range of the crystal width (stubby rod-shaped crystals). Our work provides the following main conclusions:

1. During milling the concentrations of structural OH groups and total water decreased and increased, respectively.
2. The total water content increased with the decreasing mean crystal length and width because of an increasing surface area of crystallites.
3. Water molecules were either adsorbed or structural, with their respective population ratio increasing from 0.6 for unmilled apatite to 2.3 for samples milled for 12–36 h. The adsorbed water was located in the surface hydrated layer and the structural water in the *c*-axis channels in the apatite crystal lattice.
4. The surface hydrated layer began to take form typical of nanocrystalline apatites for crystallites ca. 80 nm long and 60 nm wide. For the lowest crystal sizes it contained ca. 30 % of total apatite phosphorus.
5. It has been found that molecules of the adsorbed water are capable of moving from the surface of apatite into its crystal lattice. This process is mediated by proton transfer involving water molecules and hydroxide ions in the *c*-axis channels, as proposed by Yoder et al. (2012a). Probably, there exists also physical diffusion of water molecules to fill voids in the *c*-axis channels.
6. It was confirmed that the content of structural hydroxyl groups in nanocrystalline apatites decreases with the decreasing crystal size. For our samples this content decreased on milling by ca. 40 % in reference to stoichiometric apatite, but it has not been reduced to the level of ca. 20 %, typical of nanocrystalline biological apatite of bone, dentin, and dental cementum.
7. The reduction in the content of the structural hydroxyl groups was accompanied by a growing disorder within and around the *c*-axis channels, in accordance with the idea of Pasteris et al. (2004) and Wopenka and Pasteris (2005). We found that this disorder was caused by an uptake of the structural water into the *c*-axis apatite channels.

## Electronic supplementary material

Below is the link to the electronic supplementary material.

Supplementary material 1 (PDF 750 kb)

Supplementary material 2 (AVI 12217 kb)

Supplementary material 3 (MPG 3304 kb)
