# Supplementary material for: Solid-state MAS NMR, TEM, and TGA studies of structural hydroxyl groups and water in nanocrystalline apatites prepared by dry milling
Source: J Nanopart Res. 2013 Jul 30;15(8):1868. doi: 10.1007/s11051-013-1868-y (PMC3751289; doi:10.1007/s11051-013-1868-y)
Supplement: Supplementary file 1 — Supplementary material 1 (PDF 750 kb) [file 11051_2013_1868_MOESM1_ESM.pdf]

ONLINE RESOURCE 1

Journal of Nanoparticle Research

Solid-state MAS NMR, TEM and TGA studies of structural hydroxyl groups and water in nanocrystalline calcium apatites prepared by dry milling

Lukasz Pajchel, Waclaw Kolodziejski*

Medical University of Warsaw, Department of Inorganic and Analytical Chemistry, Banacha 1, 02-097 Warsaw, Poland

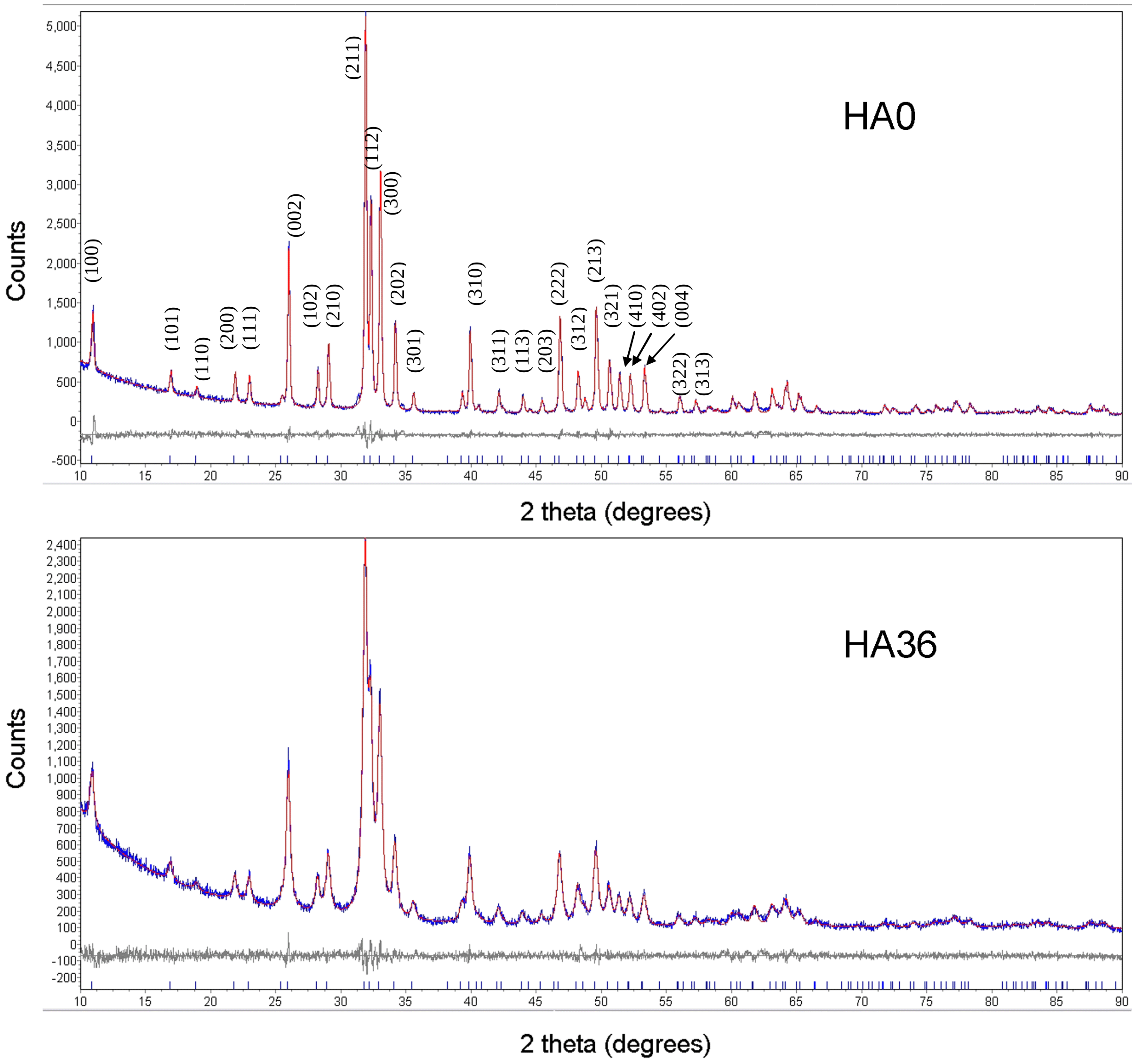

Figure 1S. Powder X-ray diffractograms of HA0 and HA36 (space group $P6_3/m$).

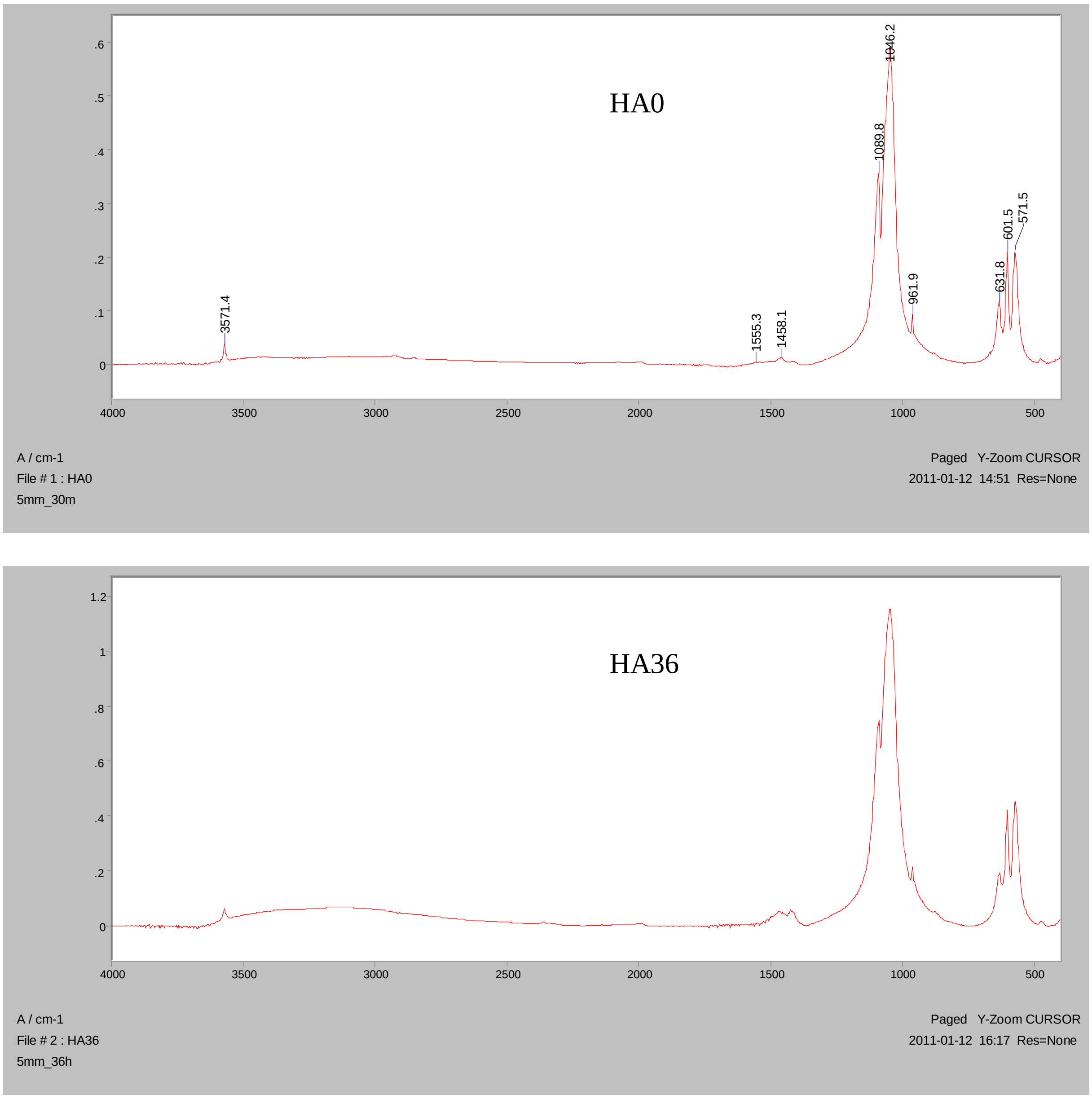

Figure 2S. FT-IR spectra of HA0 and HA36 recorded from KBr pellets.

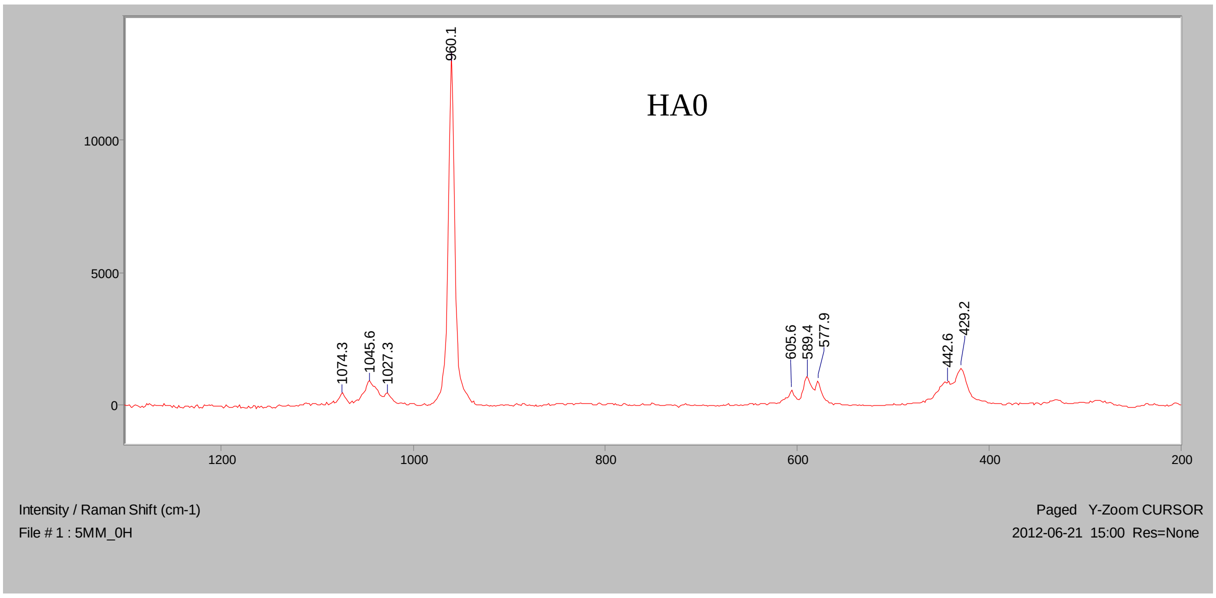

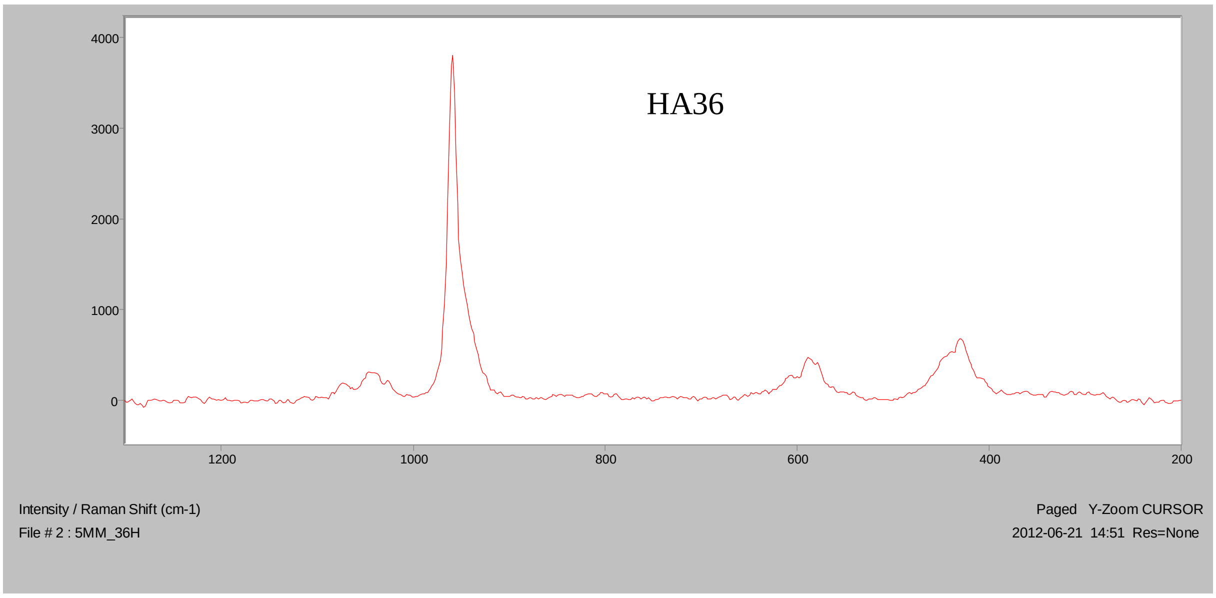

Figure 3S. Raman spectra of HA0 and HA36 recorded on an i-Raman BWS415 spectrometer of BWTEK Inc. using a 785 nm laser.

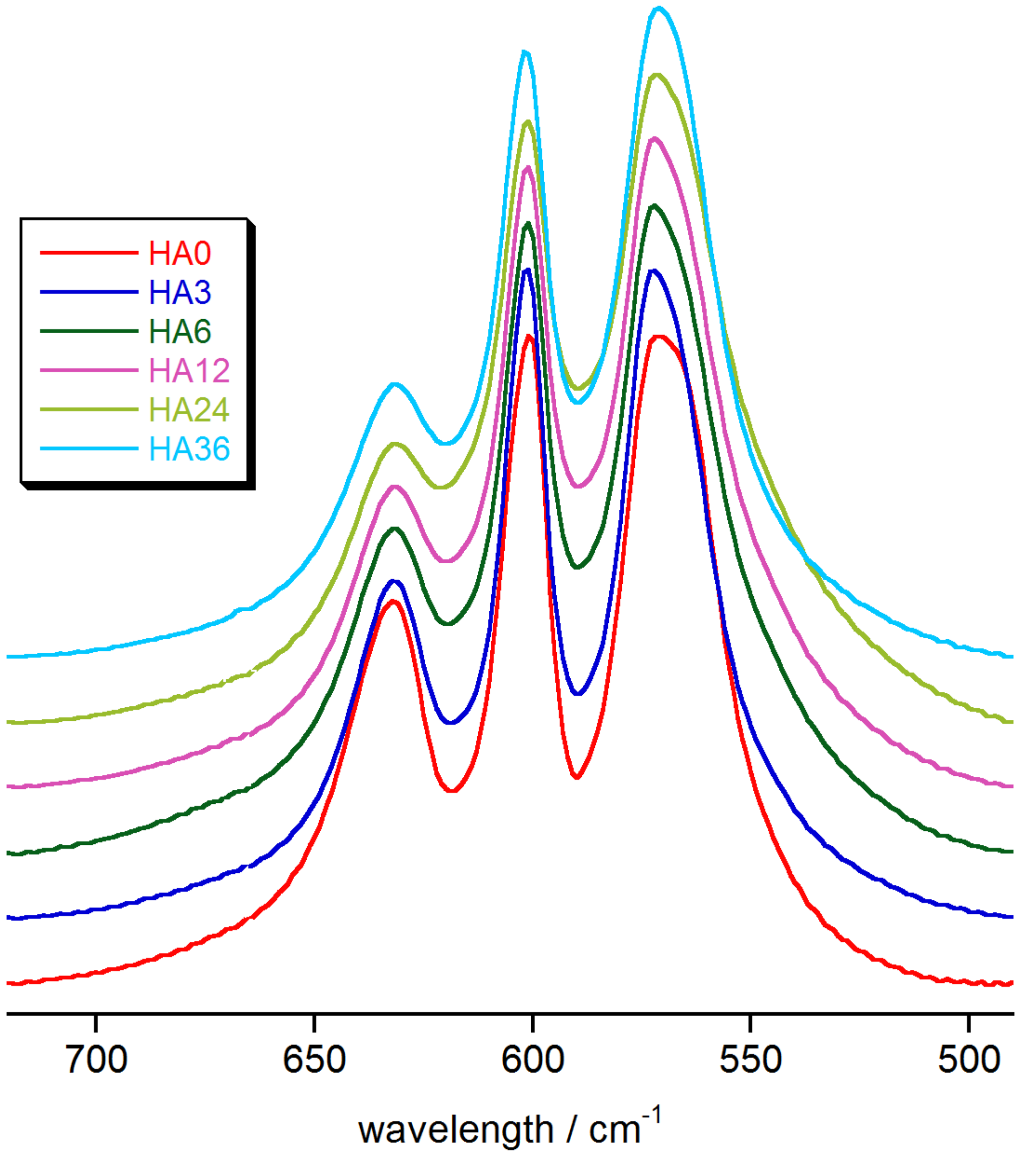

Figure 4S. FT-IR spectra in the ν_4- phosphate region.

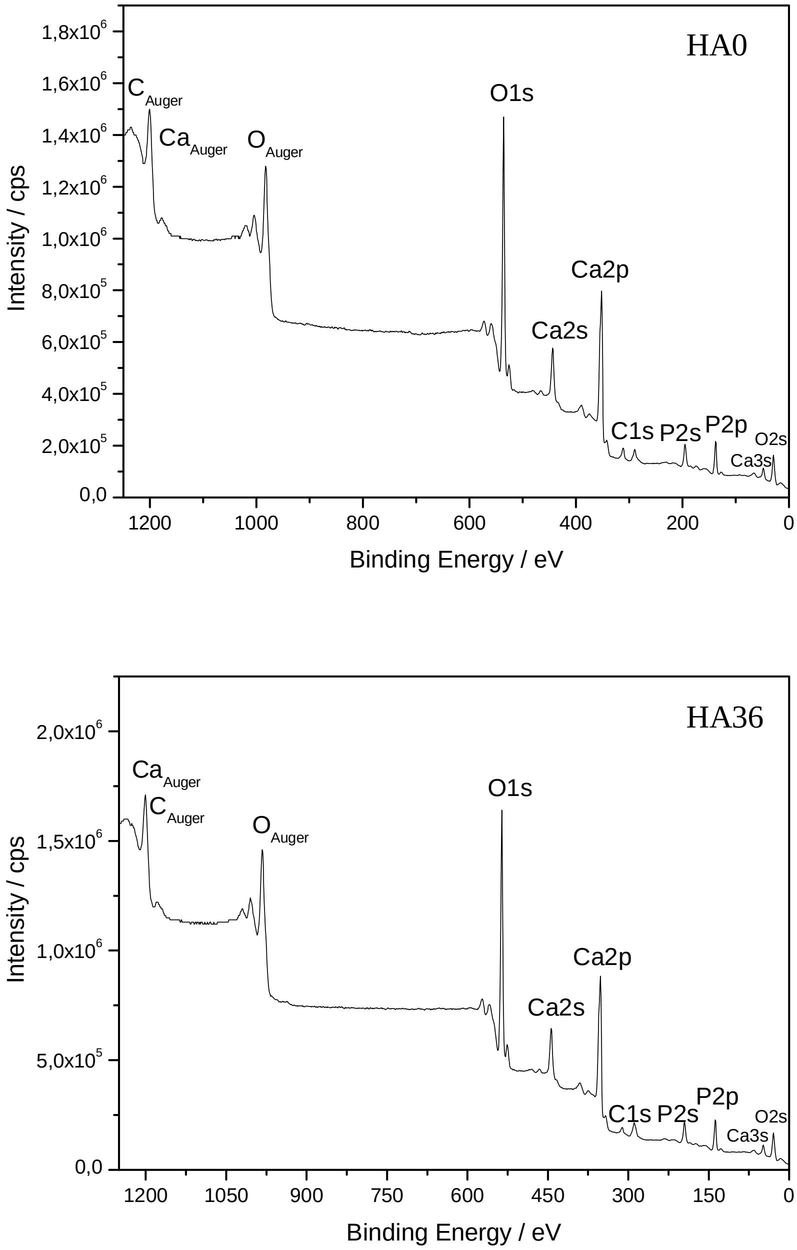

Figure 5S. XPS spectra of HA0 and HA36. The spectra show that the elemental apatite composition was not changed during sample milling.

The X-ray photoelectron studies (XPS) were done on an AES/XPS Microlab 350 spectrometer of Thermo Electron (VG Scientific). The apatite powder was fixed to an adhesive conducting tape and then analyzed with an excitation X-ray beam of energy 1487 eV ($Al_{k\alpha}$) under the maximum analyzer resolution of 0.83 eV. Full-range spectra were acquired from 0 to 1350 eV of binding energy. High-resolution spectra measured for selected chemical elements in a narrow bond energy range were referenced to the C 1s peak at 285.0 eV (the C-C chemical bond).

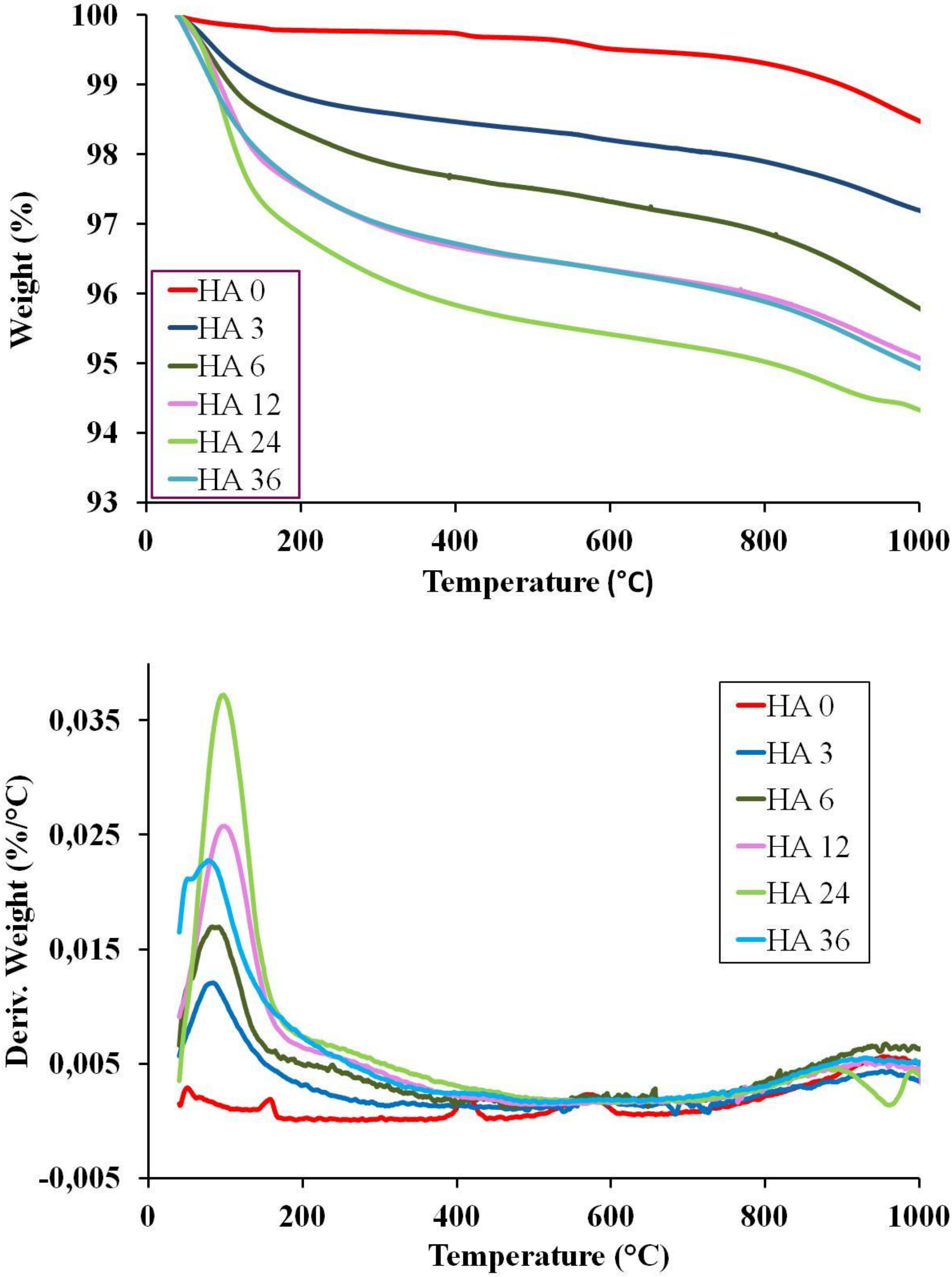

Figure 6S. TGA curves for the studied samples.

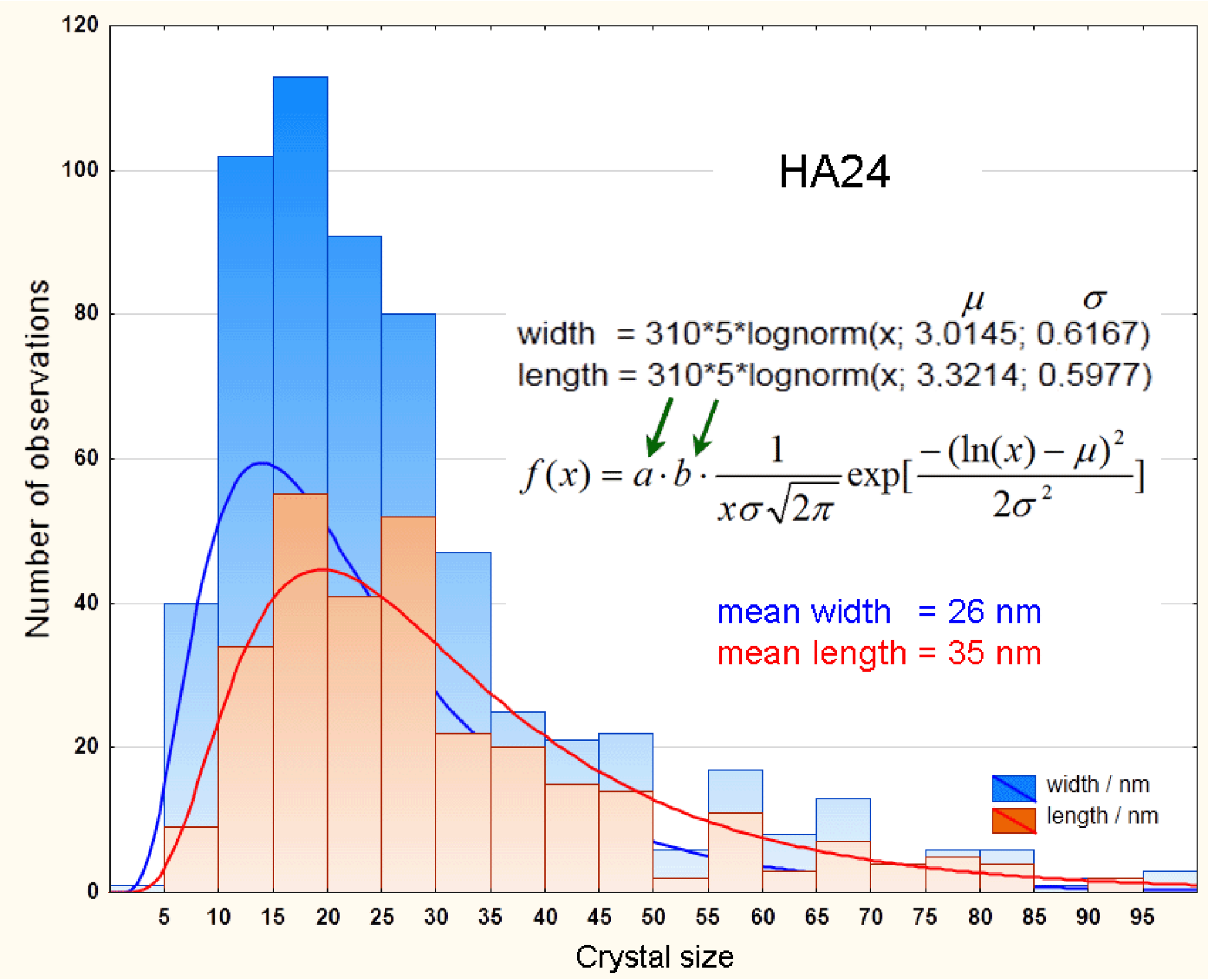

Figure 7S. Stacked histograms of crystal sizes and fitted lognormal distribution functions for HA24 (310 crystals; the STATISTICA computer program).

Table 1S. Parameters of lognormal distributions of crystal sizes performed for 310 crystals using the STATISTICA computer program.

**Sample**	**Mean length [nm]**	**μ (length)**	**σ (length)**	**Mean width [nm]**	**μ (width)**	**σ (width)**
HA0	129	4.8593	0.3747	95	4.5531	0.3804
HA0.25	97	4.5717	0.7627	71	4.2580	0.8139
HA0.5	81	4.3915	0.6909	61	4.1065	0.7328
HA1	82	4.4037	0.7077	58	4.0557	0.7370
HA2	85	4.4392	0.7645	62	4.1219	0.8034
HA3	56	4.0213	0.6701	44	3.7784	0.7098
HA4	55	4.0036	0.6397	40	3.6833	0.6673
HA5	56	4.0221	0.6008	44	3.7798	0.6213
HA6	63	4.1391	0.7110	44	3.7785	0.7062
HA12	42	3.7324	0.6670	32	3.4583	0.6865
HA24	35	3.3214	0.5977	26	3.0145	0.6167
HA36	23	3.1307	0.4707	20	2.9901	0.4728

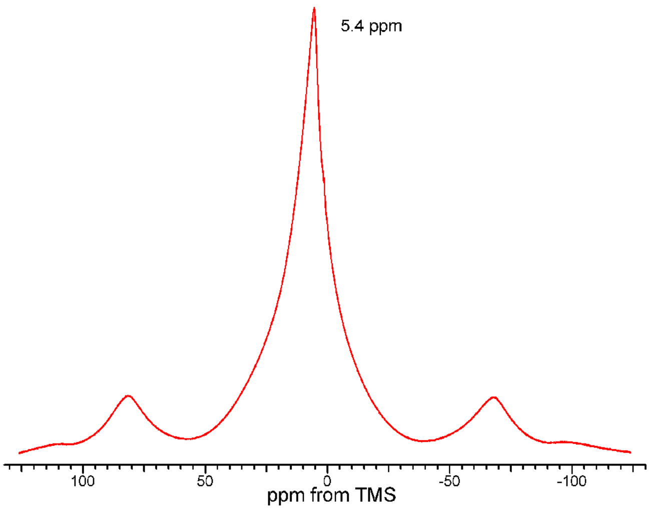

Figure 8S. Proton NMR spectrum of ACP acquired under MAS of 30 kHz.

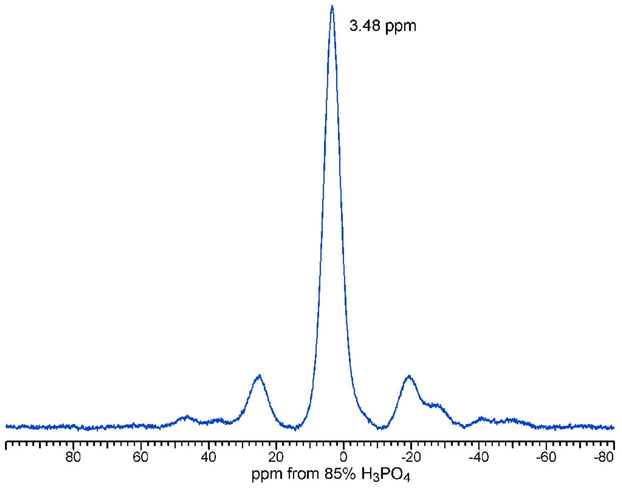

Figure 9S. ^{31}P BD NMR spectrum of ACP acquired under MAS of 3.5 kHz.

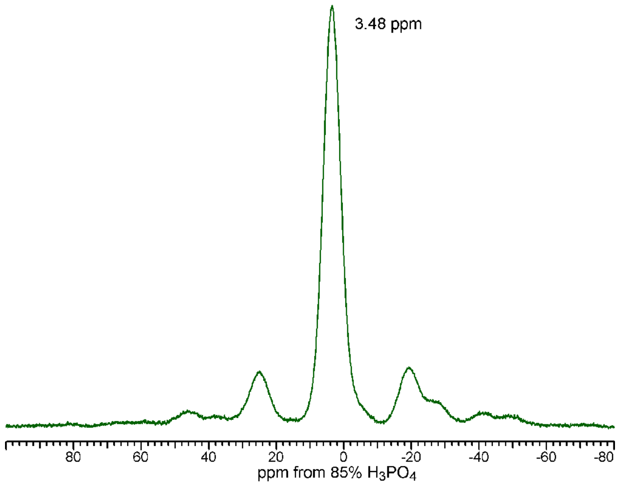

Figure 10S. ^{31}P CP NMR spectrum of ACP acquired under MAS of 3.5 kHz (contact time of 2 ms).

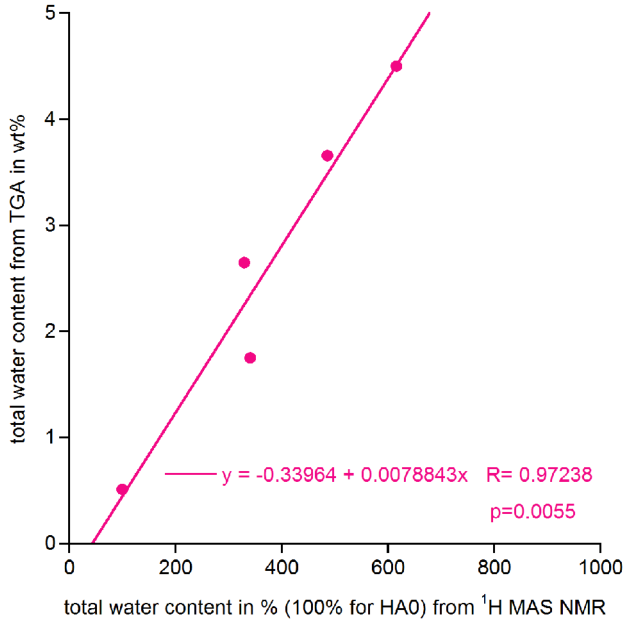

Figure 11S. Dependence of total water content calculated from TGA on total water content calculated from ^{1}H MAS NMR (MAS at 7 kHz). The outlying point of HA36 has been excluded.

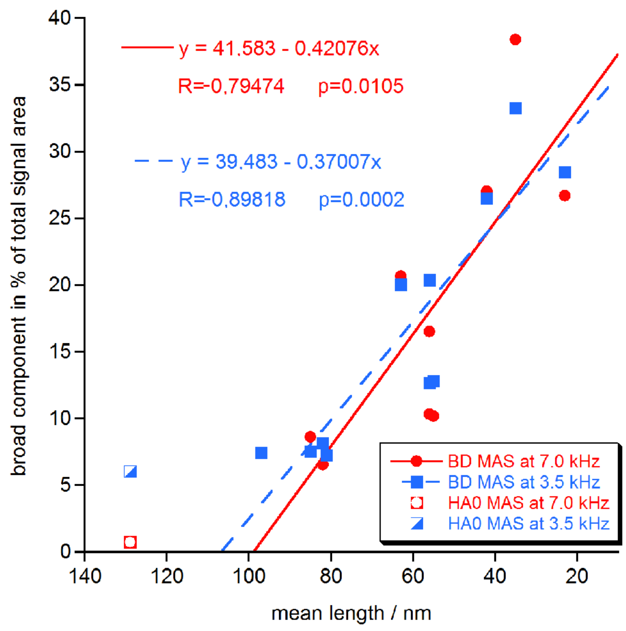

Figure 12S. Dependence of the ^{31}P BD broad component area on the mean crystal length. The outlying points of HA0 have been excluded.

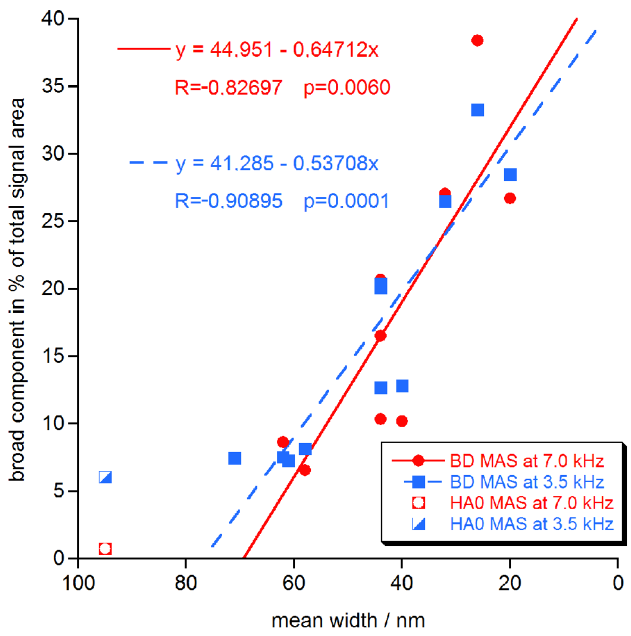

Figure 13S. Dependence of the ^{31}P BD broad component area on the mean crystal width. The outlying points of HA0 have been excluded.

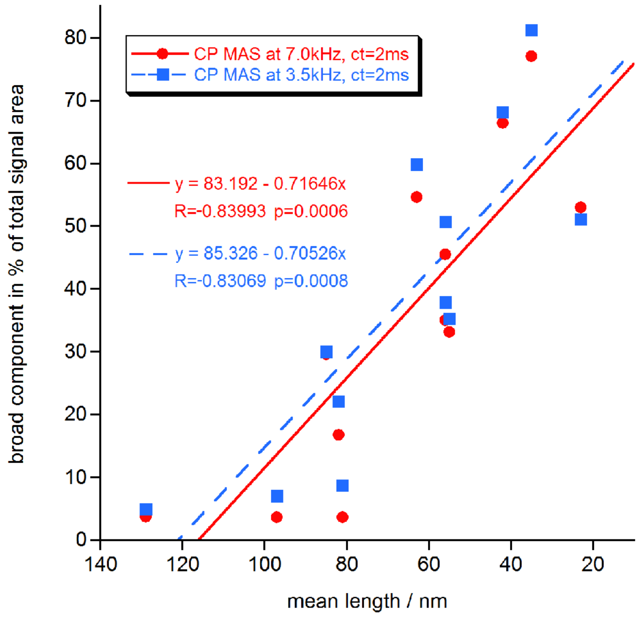

Figure 14S. Dependence of the ^{31}P CP broad component area on the mean crystal length.

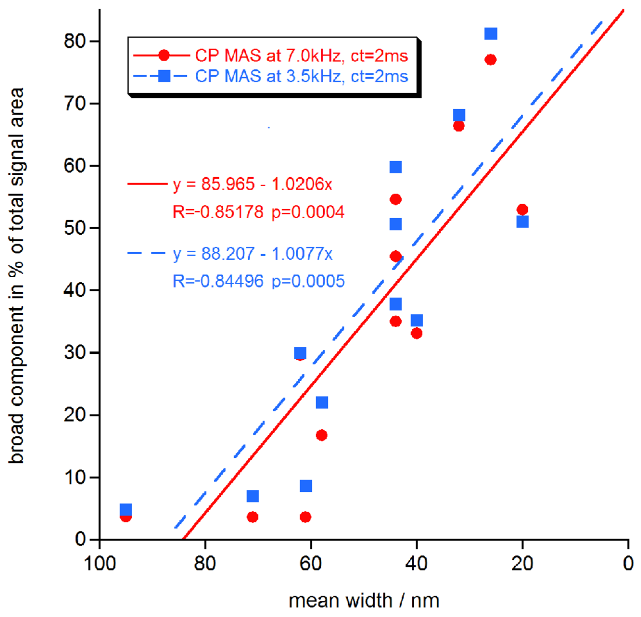

Figure 15S. Dependence of the ^{31}P CP broad component area on the mean crystal width.

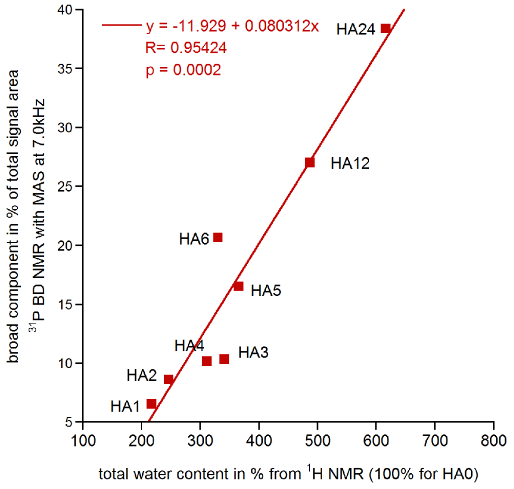

Figure 16S. Dependence of the ^{31}P BD NMR broad component area (MAS at 7 kHz) on the total water content calculated from ^{1}H NMR (without HA36).

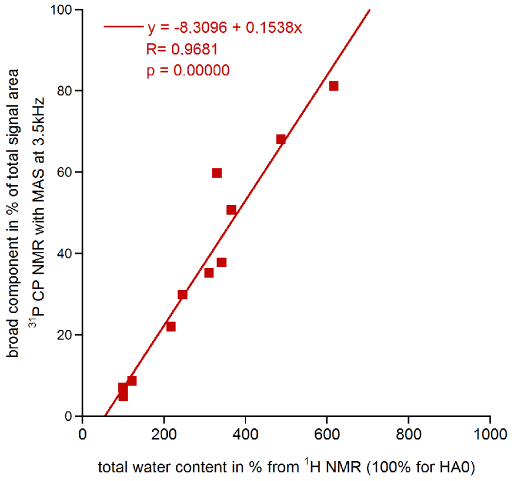

Figure 17S. Dependence of the ^{31}P CP NMR broad component area (MAS at 3.5 kHz, ct = 2 ms) on the total water content calculated from ^{1}H NMR (without HA36).

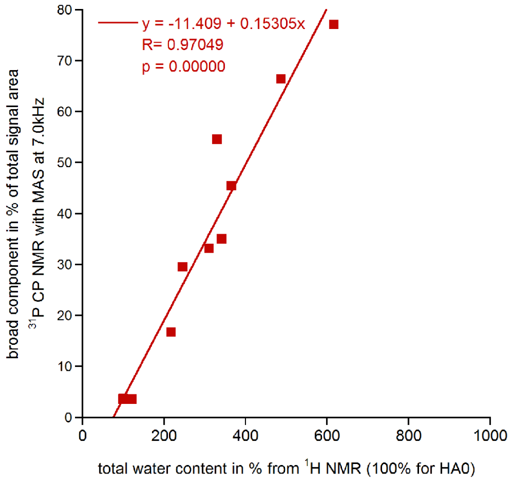

Figure 18S. Dependence of the ^{31}P CP NMR broad component area (MAS at 7 kHz, ct = 2 ms) on the total water content calculated from ^{1}H NMR (without HA36).

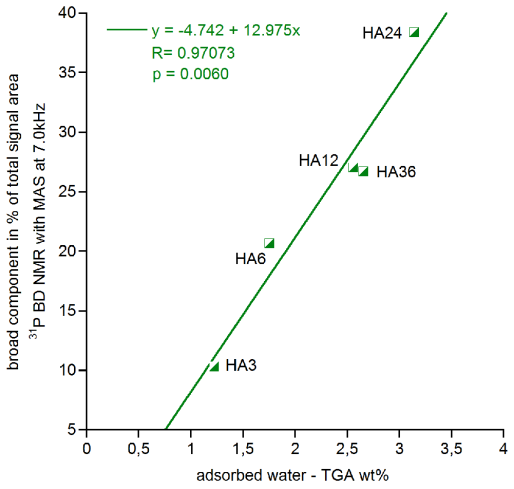

Figure 19S. Dependence of the ^{31}P BD NMR broad component area (MAS at 7 kHz) on the adsorbed water content calculated from TGA (without HA36).

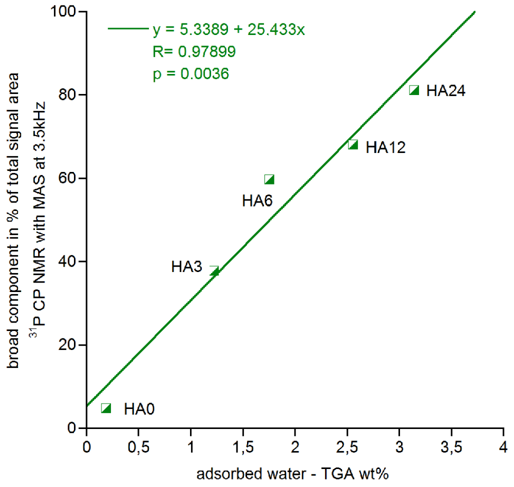

Figure 20S. Dependence of the ^{31}P CP NMR broad component area (MAS at 3.5 kHz, ct = 2 ms) on the adsorbed water content calculated from TGA (without HA36).

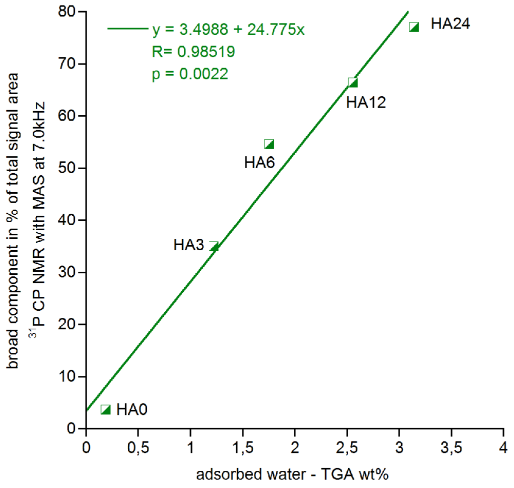

Figure 21S. Dependence of the ^{31}P CP NMR broad component area (MAS at 7 kHz, ct = 2 ms) on the adsorbed water content calculated from TGA (without HA36).

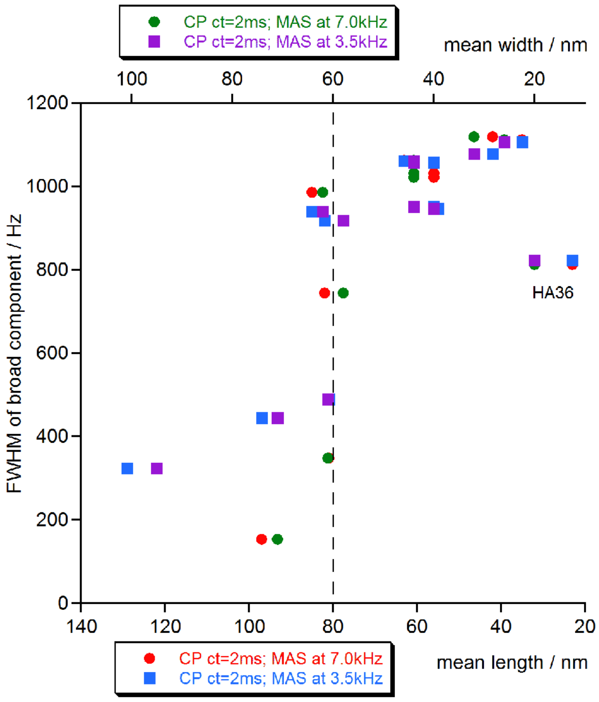

Figure 22S. Dependence of FWHM of the broad ^{31}P CP/MAS NMR component on the crystal dimensions.

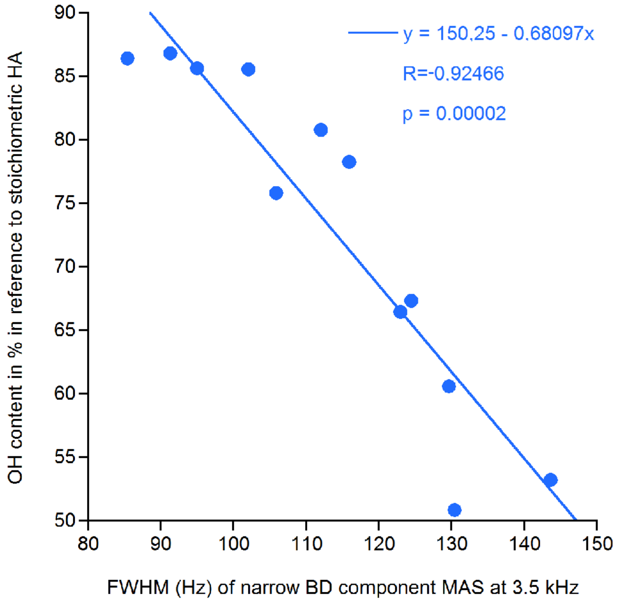

Figure 23S. Dependence of the structural OH content on FWHM of the narrow ^{31}P BD NMR component under MAS at 3.5 kHz.

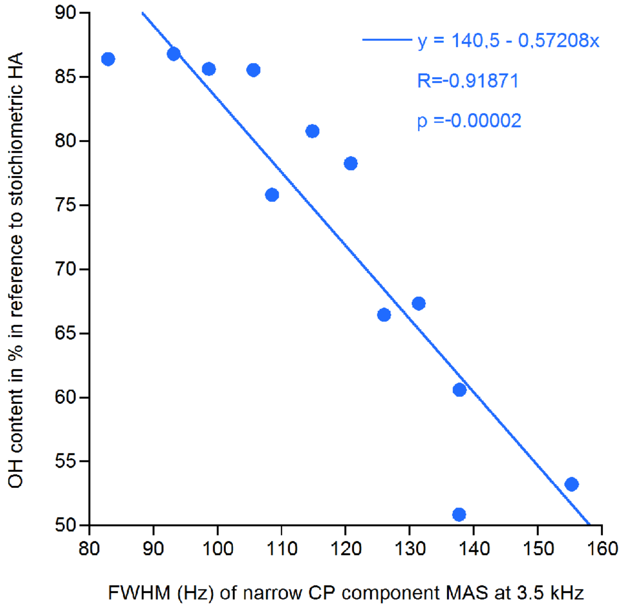

Figure 24S. Dependence of the structural OH content on FWHM of the narrow ^{31}P CP NMR component under MAS at 3.5 kHz (contact time of 2 ms).

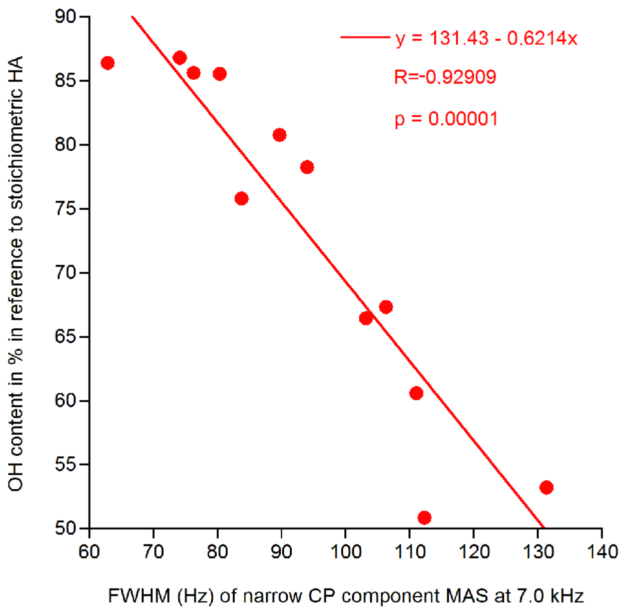

Figure 25S. Dependence of the structural OH content on FWHM of the narrow ^{31}P CP NMR component under MAS at 7.0 kHz (contact time of 2 ms).

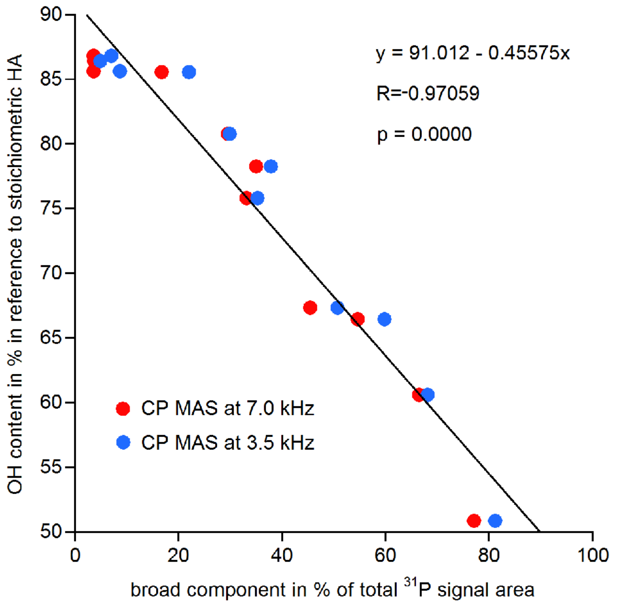

Figure 26S. Dependence of the structural OH content on the ^{31}P CP NMR broad component area (without HA36; contact time of 2 ms).
